# Combined inhibition by PRMT5 and MAT2A demonstrates a strong synthetic lethality in MTAP homozygous-deficient glioma models

**DOI:** 10.1038/s41420-025-02545-2

**Published:** 2025-05-31

**Authors:** Zuoyu Jiang, Xuetao Li, Zongyu Xiao, Wenjuan Gan, Xuewen Zhang, Yang Zhang, Weichao Wang, Qinzhi E, Yu Huang, Qikun Shi, Yi Tang, Jiaming Du, Hanmiao Dong, Jian Li, Yulun Huang

**Affiliations:** 1 Department of Neurosurgery, The Fourth Affiliated Hospital of Soochow University, Suzhou, China; 2 State Key Laboratory of Radiation Medicine and Protection, Soochow University, Suzhou, China

**Keywords:** Chemotherapy, CNS cancer

## Abstract

The intra- and intertumoral heterogeneity of gliomas present major challenges to effective chemotherapy. This study explored the combined effects of PRMT5 and MAT2A inhibitors on glioma progression. The expression of drug targets was determined in cell models using western blotting and immunofluorescence assay. CCK-8, colony-formation, EdU fluorescence, and flow cytometry cell cycle assays were conducted to assess the effect of the drugs on cell proliferation. Additionally, TUNEL fluorescence assay, flow cytometry apoptosis assay, western blotting, and comet assay were used to evaluate drug-induced apoptosis and DNA damage. Immunohistochemistry was used to validate the effect of the drugs in a 3D glioma organoid model. Patient-derived orthotopic xenograft models were used for in vivo efficacy evaluations. Lastly, transcriptome sequencing was used to elucidate the mechanism of action of the drugs, which was confirmed using western blotting. In phenotypic experiments, PRMT5 inhibitors reduced SDMA levels, inhibited cell proliferation, and promoted apoptosis in glioma models. The combination of PRMT5 inhibitors with MAT2A inhibitors enhanced synthetic lethality, leading to more potent antitumor effects. In vivo studies demonstrated that the drug combination significantly inhibited tumor growth and prolonged survival time. Our study proved the combination of PRMT5 and MAT2A inhibitors may induce synthetic lethality by downregulating the PI3K-AKT pathway, indicating the potential of this approach in treating gliomas.

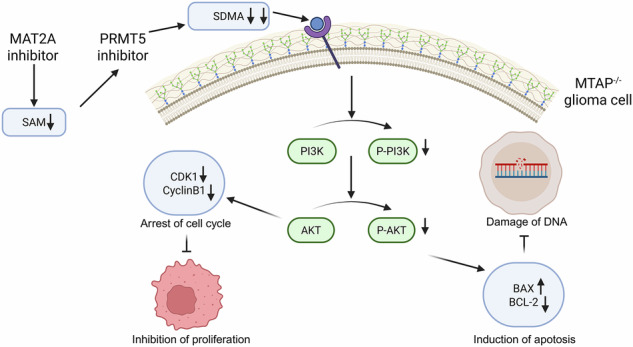

## Introduction

Glioma is a malignant tumor originating from glial cells and accounting for approximately 30% of all primary brain tumors [1]. Gliomas can be categorized into 4 grades based on the World Health Organization classification. Among these, glioblastoma multiforme (GBM) is the most aggressive and classified as grade IV. The prognosis for GBM is poor, with a median survival that is less than 15 months. Current glioma treatments rely primarily on surgical resection, radiotherapy, and chemotherapy; however, these approaches have limited efficacy and recurrence is common [2, 3]. Therefore, the identification of novel therapeutic targets and strategies is warranted.

The earliest references to synthetic lethality can be traced back to studies conducted on yeast and fruit flies [4], which have explored specific gene interactions to facilitate the understanding of developmental processes. However, the concept gained traction in cancer therapy when researchers recognized that cancer cells, particularly those with certain gene mutations such as BRCA1 and BRCA2, could be selectively targeted using specific inhibitors. A notable example is the combined lethal effect observed between BRCA1/2 mutations and PARP inhibitors in breast cancer cells [5]. PRMT5 and MAT2A show promise as a combined target in gliomas where 5′-methylthioadenosine phosphorylase (MTAP) deficiency accounts for approximately 30% [6].

The human *PRMT5* gene is located on chromosome 14, spans 8867 base pairs, and contains 18 exons that can be alternatively spliced into 6 different transcripts. The catalytic domain of the PRMT5 protein is highly conserved and consists of the following 4 well-defined domains: an N-terminal triosephosphate isomerase barrel, a central Rossmann fold domain, a C-terminal dimerization β-barrel domain [7], and a dimerization domain of approximately 60 amino acids inserted within the β-barrel domain. PRMT5 can self-associate via its N-terminal domain to form a PRMT5 tetramer, which can further specifically bind with 4 methylosome protein 50 (MEP50) molecules to form an octameric complex that initiates and enhances the enzymatic activity of PRMT5 [8].

PRMT5 is a type II protein arginine methyltransferase primarily responsible for the methylation of arginine residues to form symmetric dimethylarginine (SDMA). PRMT5 is involved in several biological processes such as cell growth, differentiation, signal transduction, and the regulation of gene expression [9]. Abnormal expression and dysfunction of PRMT5 have been reported in various tumors including breast cancer, lung cancer, and liver cancer. High PRMT5 expression in gliomas is closely associated with tumor aggressiveness. Therefore, PRMT5 is considered a potential therapeutic target to treat gliomas, and the development of PRMT5 inhibitors is gaining popularity in cancer research.

The harm due to brain cancer is evident given the absolute importance of this organ. Brain cancer is a general term for intracranial tumors that includes many subtypes, with astrocytomas and glioblastomas being the most common. Numerous studies have confirmed that PRMT5 can promote the malignant progression of gliomas through multiple pathways, indicating it to be a potential therapeutic target. PRMT5 is overexpressed in gliomas and is associated with poor prognosis. On the one hand, inhibiting PRMT5 activity can promote cell cycle arrest and the apoptosis of tumor cells, reducing its metastatic capability [10–12]; on the other hand, it can restore the expression of tumor suppressor genes such as *ST7* and *PTEN* in gliomas. One of the plausible mechanisms is that PRMT5 inhibitors can dissociate PRMT5 from the promoter region of *ST7* and restore its expression [13]. PRMT5 is an important splicing factor. Downregulating PRMT5 or inhibiting its activity can specifically disrupt the intron-removal process during mRNA precursor processing, leading to the degradation of genes related to the cell cycle and proliferation and thereby counteracting tumor growth. In 2019, Du et al. reported that PRMT5 could regulate cancer progression by affecting genomic stability in gliomas. MTAP is a tumor suppressor gene. PRMT5 activity is inhibited in MTAP-deficient glioma cells, which, in turn, downregulates the expression of E3 ubiquitin ligase SMURF2, leading to decreased stability of downstream histone H2AX and ultimately resulting in the genomic instability of tumor cells [14]. Therefore, PRMT5 may have a role in promoting genomic stability, which is consistent with the findings on PRMT5 in stem cells reported by Shinseog et al. in 2014 [15].

MAT2A has been reported to be a key synthetic lethal target in 3 independent shRNA screens performed in MTAP-deficient cell lines [16–18]. The methionine adenosyltransferase (MAT) enzyme family consists of 3 proteins and plays an important role in synthesizing S-adenosylmethionine (SAM) [19]. MAT2A converts methionine and ATP into S-adenosylmethionine (SAM) via an adenosine intermediate [20]. Next, SAM is subsequently used by methyltransferases such as PRMT5 for methylation reactions. The overexpression and increased activity of MAT2A result in the production of higher levels of SAM, which are associated with the progression of liver cancer [21], hepatocellular carcinoma [22, 23], and colorectal cancer [24, 25]. Therefore, inhibiting MAT2A can serve as a potential therapeutic approach to suppress tumor growth, especially in cancers that lack MTAP.

Considering the effects of the above targets on glioma, a series of small molecule inhibitors targeting the MAT2A–PRMT5 signal axis including a brain penetrating PRMT5-MTA inhibitor and MAT2A inhibitor [26] was recently and separately synthesized by our research group to test their effects when used alone or in combination.

## Results

### Lower MTAP expression predicts a worse prognosis, and the targets of the inhibitors are ubiquitously expressed in glioma

**Fig. 1:**
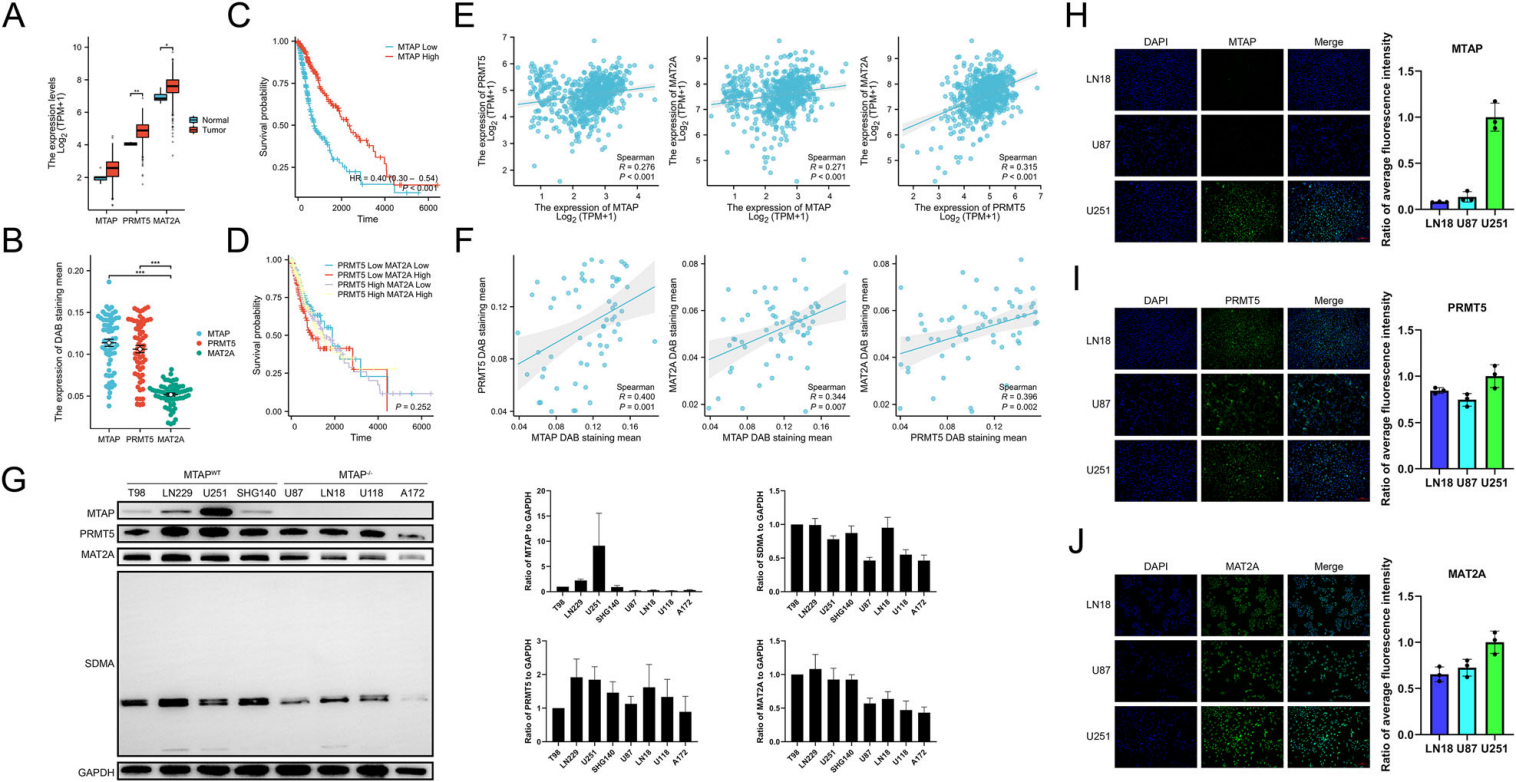
Lower MTAP expression predicts a worse prognosis. The target of the inhibitor is ubiquitously expressed in glioma and there is a co-growth correlation of expression. **A** Difference in RNA expression of *MTAP, PRMT5*, and *MAT2A* genes between tumor tissues and normal tissues in TCGA 2016 glioma database. **B** Difference in DAB staining, mean of MTAP, PRMT5, and MAT2A in tissue microarray (*n* = 61). **C** Difference in overall survival time between the 2 groups with high and low MTAP expression. **D** Differences in overall survival among the 4 groups. Each group is categorized by high and low expression of PRMT5 and MAT2A. **E** Correlation scatter plot of *MTAP, PRMT5*, and *MAT2A* genes in TCGA 2016 glioma database. **F** Correlation scatter plot of DAB staining, mean values of *MTAP, PRMT5*, and *MAT2A* genes in tissue microarray (*n* = 61). **G** Western blotting of MTAP, PRMT5, MAT2A, and SDMA in different glioma cell lines and the statistical analysis bar chart (*n* = 3). **H**–**J** Immunofluorescence to determine the expression of MTAP, PRMT5, and MAT2A in 3 glioma cell lines and the statistical analysis bar chart (scale bar = 100 μm) (*n* = 3).

Analysis of clinical data and RNA sequencing results from 668 patients from TCGA database revealed that PRMT5 and MAT2A expression was higher in tumor tissues than in normal tissues. However, MTAP expression was not significantly different (Fig. 1A).

Patient samples were collected and a glioma tissue microarray was constructed (n = 61). Tissue microarray analysis revealed that the extent of DAB staining for MTAP and PRMT5 was generally higher than that for MAT2A in glioma samples (Fig. 1B). Survival analysis of TCGA database revealed that lower MTAP expression was associated with shorter overall survival, whereas the expression of PRMT5 and MAT2A was not significantly correlated with patient prognosis (Fig. 1C, D). Consistently, database analysis indicated a proportional increase in the expression of MTAP, PRMT5, and MAT2A (Fig. 1E), a trend that was also observed in tissue microarray analysis (Fig. 1F).

Further validation using immunofluorescence and western blotting confirmed that PRMT5 and MAT2A were consistently overexpressed in glioma cell lines, supporting their potential as inhibitor targets in future studies (Fig. 1G). In contrast, MTAP expression varied across cell lines (Fig. 1H). Four MTAP-deleted cell lines and four wild-type MTAP cell lines were tested. Three cell lines with high inhibitor target expression, namely, U251 (MTAPWT), LN18 and U87 (MTAP-/-), were selected based on the differential expression of PRMT5 and MAT2A for subsequent experiments(Fig. 1I, J).

This approach sets the stage for the further investigation of the therapeutic potential of targeting PRMT5 and MAT2A in glioma.

### The PRMT5 inhibitor is more sensitive to homozygous MTAP-deleted glioma cell lines, and their combination can produce a synergistic lethal effect

**Fig. 2:**
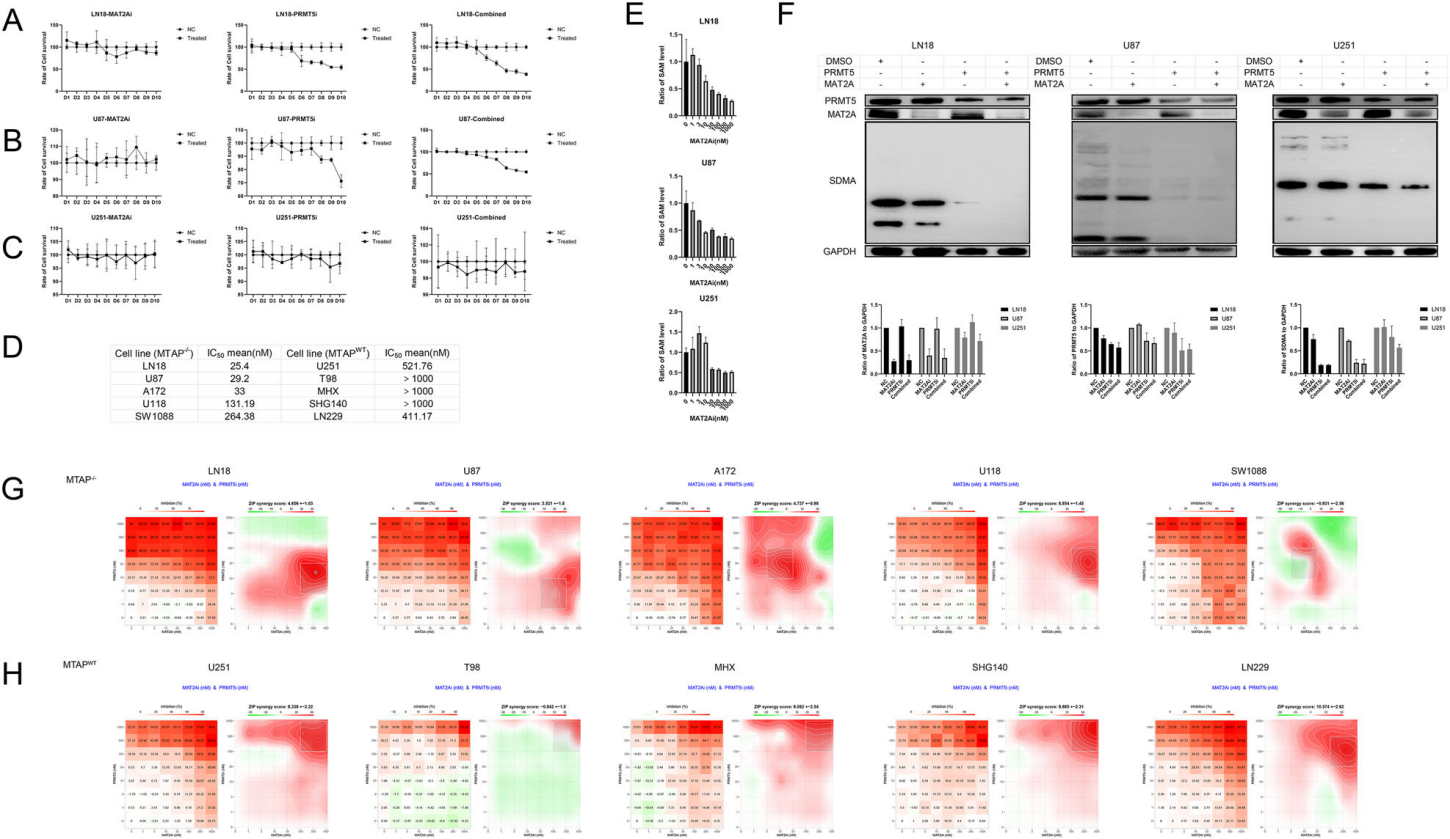
Inhibitors are more sensitive to homozygous MTAP-deleted glioma cells, and their combination can produce a stronger synergistic lethal effect. **A**–**C** CCK-8 assay to determine the viability of LN18, U87 and U251 cells after treatment with the PRMT5 inhibitor alone (10 nM), MAT2A inhibitor alone (10 nM), and with the inhibitor combination. **D** Mean IC50 values of the PRMT5 inhibitor for individual cell lines (*n* = 3). **E** LC–MS analysis of SAM levels in LN18, U87, and U251 cell lines. **F** Western blotting to determine the change trend in PRMT5, MAT2A, and SDMA in LN18, U87 and U251 cells (PRMT5i: 10 nM; MAT2Ai: 10 nM), and the statistical analysis bar chart (*n* = 3). **G**, **H** SynergyFinder 3.0 analysis for drug synergy with the PRMT5 inhibitor and MAT2A inhibitor in MTAP−/− glioma cells and MTAP+/+ glioma cells. Positive scores (red) indicate synergism, and negative scores (green) indicate antagonism (*n* = 3).

PRMT5-dependent mechanistic effects impacting cell viability, growth, proliferation, and survival were therefore observed within weeks of treatment initiation as opposed to hours (Fig. 2A, B). U251 cells showed no significant response at the same concentration (Fig. 2C). On day 8, after large-scale co-treatment using 10 cell lines, including 5 cell lines with MTAP homozygous deletions and 5 with MTAP wild-type, a CCK-8 assay was conducted. Analysis using SynergyFinder 3.0 revealed that MTAP-deleted cells were generally more sensitive to PRMT5 inhibitors when used either as monotherapy or in combination (Fig. 2D).

We further performed liquid chromatography-mass spectrometry (LC–MS) analysis to assess and validate the inhibitory effect of the MAT2A inhibitor on SAM generation in these three cell lines on 48 h. The results demonstrated that all three cell lines exhibited significant suppression of SAM production at a concentration as low as 10 nM (Fig. 2E).

The results from western blotting on day 8 confirmed that each inhibitor significantly reduced the activity of its target protein, irrespective of MTAP status. However, SDMA depletion was particularly pronounced in LN18 and U87 cells when the combination treatment was used, whereas U251 cells exhibited less SDMA depletion during the same time point (Fig. 2F). Furthermore, the combined use of the inhibitors across the 10 cell lines consistently demonstrated a synergistic lethal effect, although the concentration of the inhibitors influenced their effectiveness (Fig. 2G, H). These findings suggested that MTAP wild-type cells may require higher inhibitor concentrations or extended exposure for the inhibitory effects to be comparable.

### The combination of inhibitors can inhibit cell growth due to G2/M cell cycle arrest

**Fig. 3:**
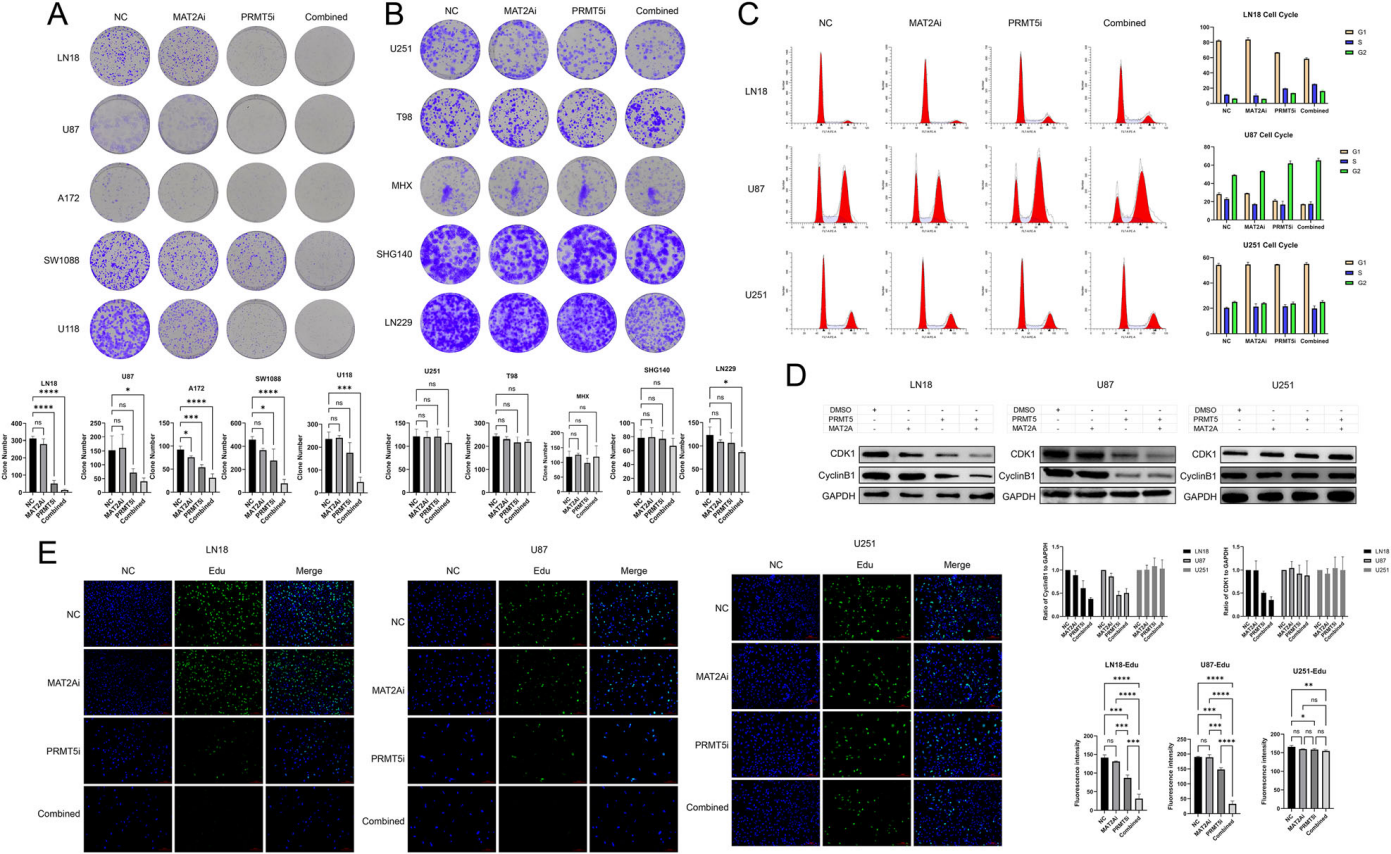
The inhibitor combination can inhibit cell growth due to G2/M cell cycle arrest. **A**, **B** Cloning experiments in 10 cell lines after treatment with inhibitors (PRMT5i: 10 nM; MAT2Ai: 10 nM), and their statistical charts (*n* = 3). One-way ANOVA for multi-group comparisons. *p < 0.05, **p < 0.01, ***p < 0.001, ****p < 0.0001. **C** Flow cytometry to determine the cell cycle in LN18, U87 and U251 cells after treatment with the inhibitors (PRMT5i: 10 nM; MAT2Ai: 10 nM), and their statistical charts (*n* = 3). (D) Western blotting to determine changes in protein expression trends of CDK1 and cyclin B1 in LN18, U87 and U251 cells (PRMT5i: 10 nM; MAT2Ai: 10 nM), and their statistical charts (*n* = 3). **E** Immunofluorescence assay to determine the effects of the 2 inhibitors (PRMT5i: 10 nM; MAT2Ai: 10 nM) in LN18, U87 and U251 cell lines using Edu staining (scale bar: 100 μm), and their statistical charts (*n* = 3). One-way ANOVA for multi-group comparisons. *p < 0.05, **p < 0.01, ***p < 0.001, ****p < 0.0001.

A plate-cloning experiment was conducted using 10 cell lines. Crystal violet staining revealed that treatment with the inhibitor combination significantly inhibited cell proliferation compared with either the single drug–treated group or the control group. This effect was particularly pronounced in homozygous MTAP-deleted cells (Fig. 3A, B).

Flow cytometry analysis of the cell cycle was performed to determine the underlying mechanism of the inhibition of cell proliferation. LN18 and U87 cells were arrested in the G2/M phase at the same inhibitor concentration, whereas U251 cells were unaffected (Fig. 3C). Cyclin B proteins (B1/B2/B3) typically bind to CDK1 during the G2 phase and trigger mitosis; however, cyclin B must accumulate to a certain threshold level for this transition to occur. Premature degradation of cyclin B halts the transition from the G2 to M phase, thereby disrupting the cell cycle [27, 28]. Western blotting further revealed a downward trend in the expression of CDK1 and cyclin B1 proteins (Fig. 3D).

To further validate these findings, EdU immunofluorescence staining was performed, which confirmed a similar inhibitory trend related to cell proliferation as that seen in the plate-cloning experiment. The combination therapy exhibited the greatest inhibitory effect on LN18 and U87 cells, whereas no significant changes were observed in U251 cells (Fig. 3E).

Overall, these findings suggested that inhibition of cell growth in homozygous MTAP-deleted cells was likely associated with G2/M cell cycle arrest.

### The combination of inhibitors can cause apoptosis due to DNA damage

**Fig. 4:**
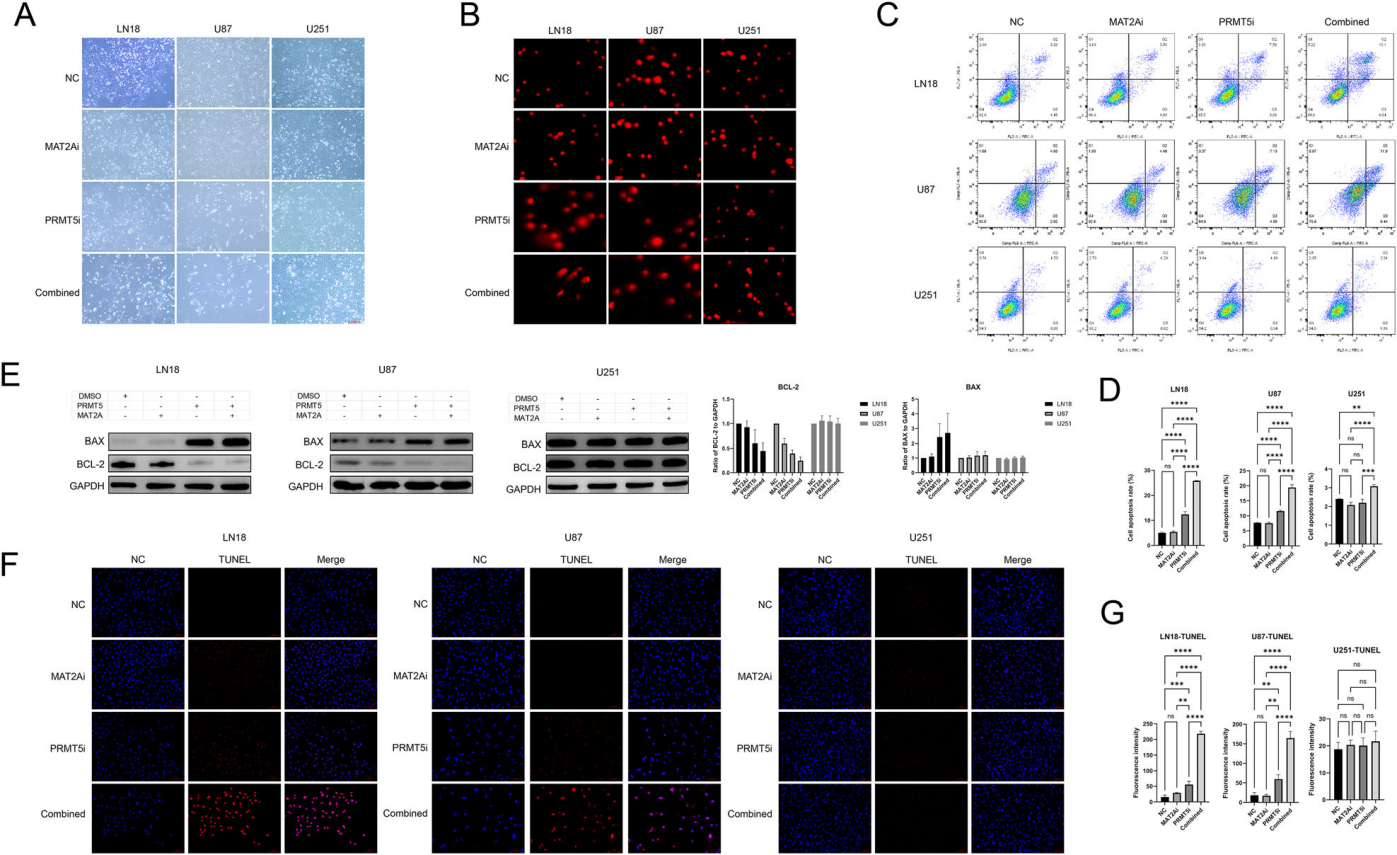
The inhibitor combination can cause apoptosis in cells via DNA damage. **A** Brightfield of LN18, U87 and U251 cell lines after treatment with the 2 inhibitors (PRMT5i: 10 nM; MAT2Ai: 10 nM) (scale bar: 100 μm). **B** Comet assay in LN18, U87 and U251 cell lines after treatment with the 2 inhibitors (PRMT5i: 10 nM; MAT2Ai: 10 nM) (scale bar: 100 μm). **C**, **D** Flow cytometry to determine the number of apoptotic cells in LN18, U87 and U251 cells after inhibitor treatment (PRMT5i: 10 nM; MAT2Ai: 10 nM), and their statistical charts (n = 3). One-way ANOVA for multi-group comparisons. *p < 0.05, **p < 0.01, ***p < 0.001, ****p < 0.0001. **E** Western blotting to determine changes in protein expression trends of BAX and BCL-2 in LN18, U87 and U251 cells (PRMT5i: 10 nM; MAT2Ai: 10 nM), and their statistical charts (*n* = 3). **F**, **G** Immunofluorescence assay to determine the effects of the 2 inhibitors (PRMT5i: 10 nM; MAT2Ai: 10 nM) in the LN18, U87 and U251 cell lines using TUNEL staining (scale bar: 100 μm), and their statistical charts (*n* = 3). One-way ANOVA for multi-group comparisons. *p < 0.05, **p < 0.01, ***p < 0.001, ****p < 0.0001.

Cell morphology was studied using light microscopy. LN18 cells in both the combination and PRMT5 inhibitor-treated groups showed signs of shrinkage as well as a significant amount of cell debris in the extracellular medium (Fig. 4A). Additionally, the results from comet electrophoresis demonstrated a higher extent of DNA damage in the group treated with the inhibitor combination versus that in the other groups (Fig. 4B).

The findings were consistent with the results obtained from flow cytometry for apoptosis determination, wherein the percentages of early and late apoptotic cells were markedly higher in the combination group than in the other groups (Fig. 4C, D).

Western blotting of apoptosis-related proteins further supported these findings, revealing an increase in BAX levels and a decrease in BCL-2 levels after treatment with the inhibitor combination (Fig. 4E).

Immunofluorescence and TUNEL assay also revealed that LN18 and U87 cells treated using the inhibitor combination exhibited a significantly higher number of TUNEL-positive cells compared with LN18 and U87 cells treated using the PRMT5 inhibitor alone. Treatment with the PRMT5 inhibitor showed stronger effects compared with that achieved in the control and MAT2A inhibitor–treated groups, and these differences were statistically significant (Fig. 4F, G).

Interestingly, these effects were not observed in U251 cells in other experiments, suggesting that the combination of PRMT5 and MAT2A inhibitors induced significant DNA damage and apoptosis specifically in homozygous MTAP-deleted cells.

### The combination of PRMT5 and MAT2A inhibitors results in synthetic lethal effects in glioma organoid models

**Fig. 5:**
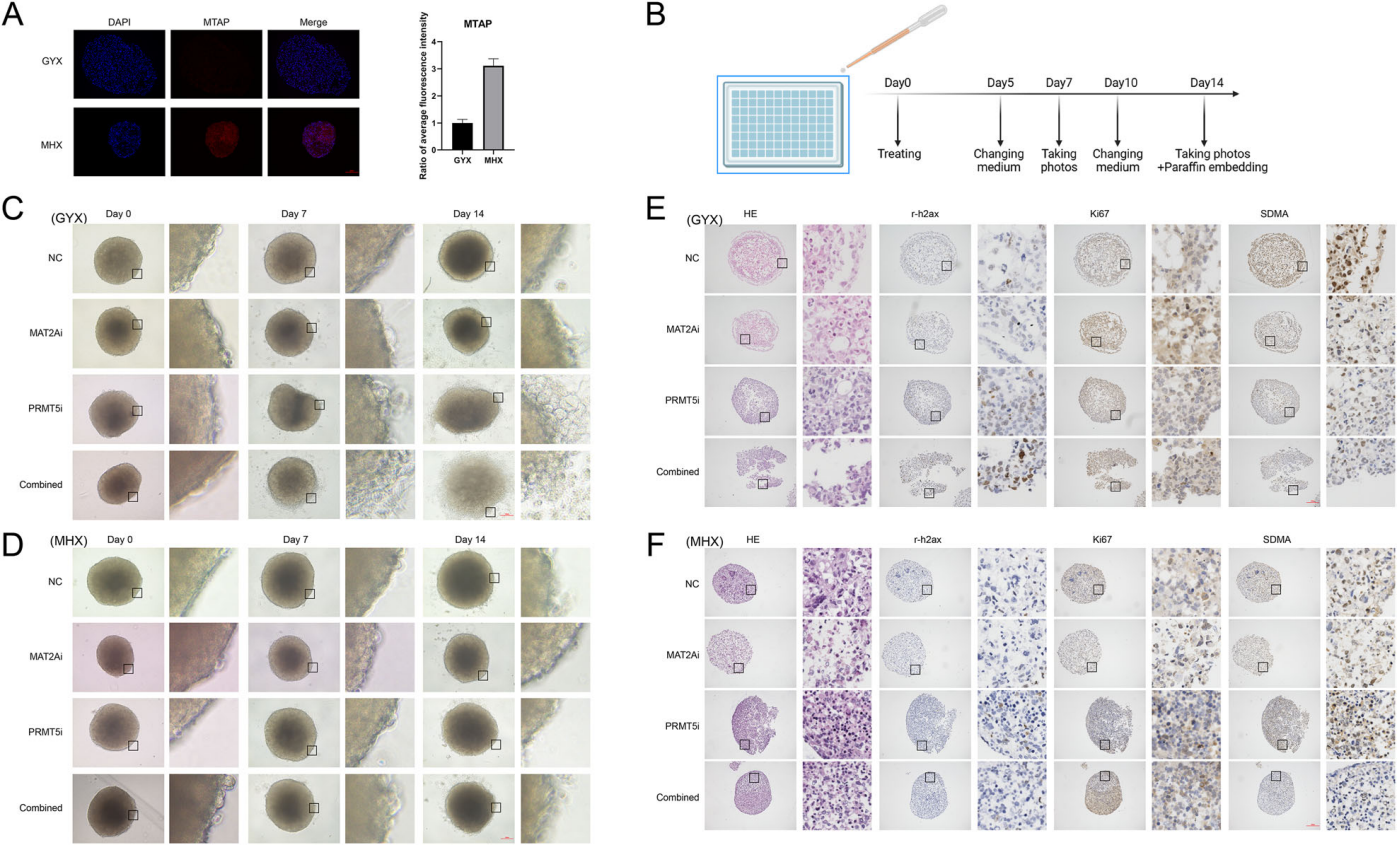
The combination of PRMT5 and MAT2A inhibitors produces synthetic lethal effects in glioma organoid models. **A** Immunofluorescence assay to determine MTAP expression in 2 glioblastoma organoids, and their statistical charts (scale bar: 200 μm) (*n* = 3). **B** Flowchart of organoid experiments. **C**, **D** Brightfield images showing the cellular state of organoids after treatment with the inhibitors at 7 days and 14 days (scale bar: 200 μm). **E**, **F** H&E staining and immunohistochemical staining of SDMA, Ki67, γ-H2AX using paraffin sections of glioblastoma organoids after 14 days of co-culture (scale bar: 200 μm).

After constructing glioma organoids from patients and verifying their parental characteristics, MTAP expression was tested using immunofluorescence experiments. Finally, 2 cell lines with high inhibitor target expression, namely, PDO-GYX (MTAP^−/−^) and MHX (MTAP^WT^), were selected based on their activity and stability (Fig. 5A).

Several parallel cultures were conducted after selecting the organoids, and organoids of similar sizes were chosen from the same passage for subsequent experiments. The organoids were placed in 96-well plates and divided into the following 4 groups: control (dimethylsulfoxide), 100 nM PRMT5 inhibitor, 100 nM MAT2A inhibitor, and combination therapy groups. The medium containing the drugs was refreshed every 5 days, and light microscopy images were captured on 0, 7, and 14 days after treatment (Fig. 5B).

Light microscopy revealed that peripheral cells of the PDO-GYX organoids were mainly intact with minimal dissociation and the structures were well preserved by day 7 in the monotherapy groups. In contrast, partial shedding of peripheral cells was noted in the organoids in the combination therapy group. By day 14, the control group and MAT2A inhibitor group showed no significant cellular dissociation; however, the internal structure of the organoids appeared denser due to growth. Substantial peripheral cell shedding and structural damage were noted in the PRMT5 inhibitor group. The combination group showed the most pronounced effects with complete peripheral cell dissociation and loss of structural integrity. At the same dose, the MTAP wild-type organoids (PDO-MHX) showed no significantly visible changes by day 14 (Fig. 5C, D).

The organoids that were cultured for 14 days were fixed in 4% paraformaldehyde, embedded in paraffin, and subjected to immunohistochemical staining. H&E staining of PDO-GYX organoids revealed that the organoids in the PRMT5 inhibitor group exhibited partial cytoplasmic condensation, pronounced chromatin clumping, increased γ-H2AX expression (a marker of DNA damage), reduced Ki67 (a proliferation marker) staining, and a marked reduction in SDMA (a methylation marker) staining. These effects were even more pronounced in the organoids in the combination therapy group, where the overall structure showed significant damage. In contrast, PDO-MHX organoids showed no notable damage and no significant changes in γ-H2AX or Ki67 expression. However, SDMA depletion was noted, which was in alignment with the previous findings from the cell models (Fig. 5E, F).

### Inhibitors can significantly improve the survival time and reduce the tumor burden in vivo in nude mice

**Fig. 6:**
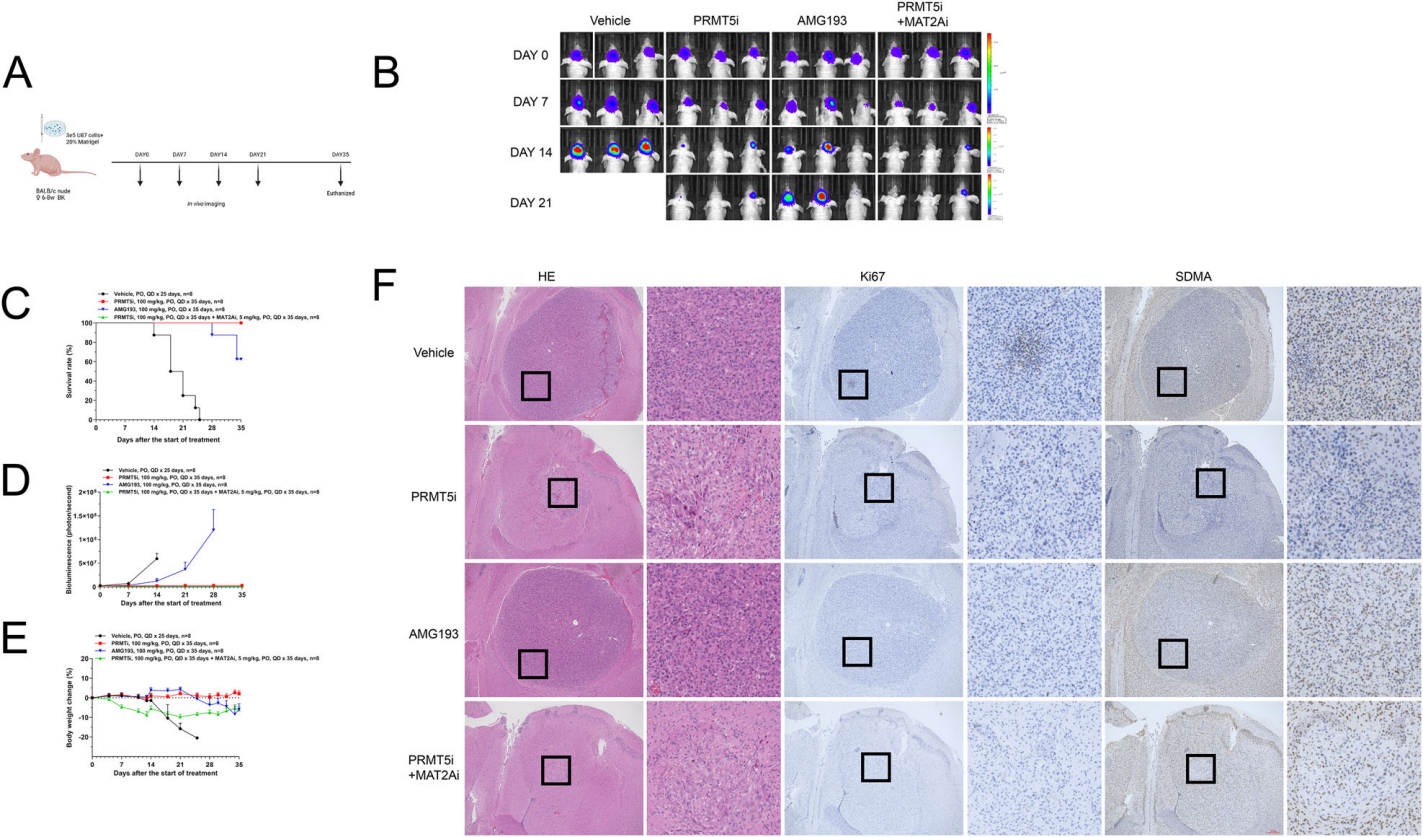
Inhibitors can significantly improve survival time and reduce tumor burden in nude mouse models in vivo. **A** Flowchart of animal experiments. **B** Representative bioluminescence images of 4 groups of mice injected with cells (Vehicle, AM193 [100 mg/kg, PO, QD], PRMT5 inhibitor [100 mg/kg, PO, QD], combination of the PRMT5 inhibitor [100 mg/kg, PO, QD] and MAT2A inhibitor [5 mg/kg, PO, QD]). **C** Survival analysis of the 4 groups. **D** Bioluminescence analysis of the 4 groups. **E** Body weight analysis of the 4 groups. **F** H&E staining and immunohistochemical staining (Ki67, SDMA) of the brain sections of mice (scale bar: 100 μm).

A mouse model of orthotopic glioma was established using 3 × 10^5^ U87 cells per nude mouse (*n* = 32) (Fig. 6A). After successful establishment of the in vivo model, mice were randomly divided into the following 4 groups: vehicle, AM193: a clinical stage MTA-Cooperative PRMT5 inhibitor [29] (100 mg/kg, oral [PO], once daily [QD]), PRMT5 inhibitor (100 mg/kg, PO, QD), and the combination of a PRMT5 inhibitor (100 mg/kg, PO, QD) and MAT2A inhibitor (5 mg/kg, PO, QD) groups.

All control mice succumbed to the tumor burden by day 25 (Fig. 6B, C). However, mice in the inhibitor-treated groups exhibited varying degrees of tumor suppression. Luciferase luminescence imaging was used to monitor the tumor response. Mice in the AMG193 and control groups showed poor tumor suppression, whereas those in the PRMT5 inhibitor group exhibited substantial tumor inhibition. Mice in the combination therapy group demonstrated the most significant tumor reduction (Fig. 6D). Importantly, no significant weight loss was noted in mice in any treatment group, indicating low toxicity (Fig. 6E).

Paraffin sections were stained and weak inhibitory effects on tumor growth were noted in both the control and AMG193 groups. In contrast, a significant reduction in tumor size was noted in the PRMT5 inhibitor group, and the combination treatment group exhibited stronger inhibitory effects. H&E staining indicated a decrease in the density of tumor tissues, whereas immunohistochemical analysis showed a marked reduction in the expression of Ki67 and SDMA, confirming the synergism of the combination therapy (Fig. 6F).

### Transcriptome sequencing suggests inhibition of the PI3K-AKT pathway after treatment

RNA sequencing followed by KEGG and GO analyses revealed significant alterations in RNA expression after treatment with the 2 inhibitors (Supplementary Fig. 1A). Several genes were upregulated or downregulated (Supplementary Fig. 3), and several tumor-related pathways were activated. The most affected genes were associated with extracellular matrix structure, cell adhesion, and signal transduction, which were in alignment with the dissociation of PDO cells observed after treatment (Supplementary Fig. 1B).

KEGG analysis was used to further analyze the PI3K-AKT pathway, a critical regulator of tumor growth (Supplementary Fig. 1C). The results from western blotting further confirmed that this pathway was significantly inhibited after treatment with the inhibitors (Supplementary Fig. 1D).

To further reverse-validate that the drug caused cell death through the PI3K-AKT pathway, the AKT pathway activator SC79 (10 μM) [30] was added to cell proliferation–related experiments (CCK-8 assay, plate cloning and Edu staining) and apoptosis-related experiments (TUNEL staining and flow cytometry) as described previously. At the cellular proliferation level, this pathway activator partially reversed the synthetic lethal effect, leading to an increase in cell viability and colony formation (Supplementary Fig. 2A, B). In terms of apoptosis, it reduced both early and late apoptotic cells (Supplementary Fig. 2C). Furthermore, EdU and TUNEL staining assays further confirmed its partial reversal effect (Supplementary Fig. 2D–G). The activator of the SC79 AKT pathway did indeed reverse the synergistic lethal effect of the PRMT5 inhibitor and MAT2A inhibitor in homozygous MTAP-deleted cells, achieving the partial reversal of inhibition of proliferation and the promotion of apoptosis.

## Discussion

Several PRMT5 inhibitors have demonstrated a notable antitumor effect in various tumor models, with key examples being GSK3326595 and JNJ-64619178 [31, 32]. These inhibitors competitively bind to the SAM-binding site of PRMT5, effectively inhibiting its methyltransferase activity, which, in turn, reduces tumor cell proliferation and survival.

GSK3326595, in particular, shows high specificity for the PRMT5/MEP50 complex, disrupting its methyltransferase function. This disruption impacts critical processes such as RNA processing, transcription, and translation, ultimately altering gene-related methylation modifications and maintaining RNA homeostasis. Furthermore, GSK3326595 induces cell cycle arrest at the G1 phase and promotes cancer cell death by increasing the expression of cell cycle–related genes, inhibiting oncogene expression, and reactivating tumor suppressor genes. In lymphoma cell lines, GSK3326595 induces exon 6 deletion in the MDM4 oncogene, reversing its inhibitory effect on the p53 pathway and restoring antitumor effect. Similarly, other small-molecule inhibitors such as EPZ015666 also show promising antitumor effects, which have been validated in preclinical studies in animal models [33].

The PRMT5 inhibitors discussed earlier are first-generation compounds. GSK3326595 and JNJ-64619178 have each progressed through clinical trials, with the former reaching phase II trials and the latter currently being in phase I trials. Both inhibitors can partially delay tumor progression, indicating their therapeutic potential. However, the clinical development of these inhibitors has been temporarily halted due to concerns about blood-related toxicity. The efficacy of these first-generation inhibitors does not appear to be linked to MTAP deficiency. In contrast, the second-generation PRMT5-MTA inhibitors developed by our team show clear selectivity for MTAP-deficient cells. When used in combination with the MAT2A inhibitors designed by our study group, these second-generation inhibitors exerted a stronger, synthetic lethal effect, which was suggestive of a more targeted and potent therapeutic strategy.

Therapies involving the combination of PRMT5 inhibitors and other agents have demonstrated strong cytotoxicity in in vitro models of glioblastoma. For example, the combination of the PP2A protein phosphatase inhibitor LB100 with a PRMT5 inhibitor has been reported to significantly reduce tumor size and prolong survival in animal models compared with monotherapy using LB100 [34]. This finding underscores the potential synergistic effects of the combination in treating glioblastoma. Furthermore, several studies have reported enhanced efficacy when a combination of PRMT5 inhibitors and other treatment modalities, such as radiotherapy, are used, with the end result being a further improvement in overall treatment outcomes. Collectively, these findings indicate the potential of combination therapies with PRMT5 inhibitors and highlight their potential in transforming strategies directed toward glioblastoma treatment.

When using the combination therapy, inhibition of the proliferation of the glioma cell line or promotion of apoptosis with the PRMT5 inhibitor led to a delayed onset of action. Consequently, significant time was spent in the early stages of this study to optimize the concentration of the inhibitor and the treatment duration. Based on the findings from the CCK-8 assay and western blotting, it was speculated that the drug may significantly reduce SDMA levels in MTAP-deficient cell lines after 8 days of treatment. Accordingly, a nanomolar concentration and an 8-day treatment period were selected for further validation. While the MAT2A inhibitor alone did not significantly inhibit cell proliferation or induce apoptosis, its ability to suppress SAM levels was confirmed using LC-MS. The PRMT5 inhibitor required more time to take effect in the 3D glioma organoid model, with the organoid structure collapsing from the outermost cells inward, suggesting the gradual penetration of the drug. These findings were consistent with the results from our in vitro and in vivo experiments. Overall, the results from the proliferation and apoptosis assays and RNA sequencing demonstrated that the combination of PRMT5 and MAT2A inhibitors induced significant synthetic lethal effects both in vivo and in vitro. Our findings provide a theoretical basis and preliminary guidance on the dosage, onset time, and evaluation period for potential future clinical trials.

PRMT5 inhibitors are a promising class of novel antitumor agents that offer several advantages. These inhibitors demonstrate high specificity and selectivity by targeting the methyltransferase activity of PRMT5, effectively suppressing tumor cell proliferation and survival. Moreover, they show significant antitumor effects and synergistic potential in combination with existing therapies, thereby enhancing the overall treatment efficacy. However, challenges such as potential side effects, drug resistance, and pharmacokinetic limitations must be addressed to optimize the efficacy and safety of these compounds. Future research should prioritize the structure optimization and performance of these inhibitors, explore effective combination therapies, and conduct rigorous preclinical and clinical trials to further validate their antitumor effects and safety, particularly in glioblastoma and other cancers.

## Materials and methods

### Culture of cell lines and PDOs, synthesis and identification of inhibitors

U87, U251, LN229, and T98 cell lines, the primary cell line SHG140, patient-derived organoids GYX and MHX from GBM samples were obtained from the Fourth Affiliated Hospital of Soochow University, Suzhou 215000, China. The cells were cultured and identified using short tandem repeat DNA profiling. LN18, A172, and U118 cells were obtained from Suzhou Genhouse Bio Co., Ltd. All cells were cultured in Dulbecco’s Modified Eagle Medium (DMEM)/F12 medium (Corning, Cat. No. 10092025) supplemented with 10% fetal bovine serum (VivaCell, Cat. No. C04001-500). All PDOs were cultured in a medium containing 235 mL of DMEM/F12 medium, 235 mL of neurobasal medium (Thermo Fisher Scientific, Cat. No. 21103049), 5 mL of MEM-NEAA solution (Thermo Fisher Scientific, Cat. No. 11140050), 5 mL of GlutaMAX supplement (Thermo Fisher Scientific, Cat. No. 35050061), 5 mL of penicillin-streptomycin solution (Thermo Fisher Scientific, Cat. No. 15070063), 5 mL of N2 supplement (Thermo Fisher Scientific, Cat. No. 17502048), 10 mL of B27 minus vitamin A supplement (Thermo Fisher Scientific, Cat. No. 12587010), and 125 μL of human recombinant insulin (Merck, Cat. No. I9278) [35]. The brain-penetrating PRMT5-MTA inhibitor and MAT2A inhibitor were synthesized and characterized by Suzhou Genhouse Bio Co., Ltd.

### Bioinformatics analysis

MTAP, PRMT5, and MAT2A expression was analyzed and their correlation expression analysis in different glioma samples was conducted using The Cancer Genome Atlas (TCGA) database [36]. Furthermore, relevant prognostic analyses were performed for these groups showing different expression.

### Cell counting kit‑8 (CCK‑8) and colony-formation assay

Assay plates for viability studies were prepared and cells were trypsinized and resuspended in fresh media. Viable cells were diluted using complete growth media and seeded in 96-well plates at a density of 500 cells per well. After treating the cells with the compound for 8 days, 10 μL of CCK-8 reagent (Dojindo, Cat. No. CK04) was added to each well, followed by incubation at 37 °C for 3 h. The absorbance was measured at 450 nm. For the colony-formation assay, cells were seeded into 6-well plates at a density of 800 cells per well and plated with 2 mL of the cell culture medium. The cells were treated with the compound for 14 days and the medium was replaced every 5 days. After 14 days, the cell colonies were fixed with 4% paraformaldehyde (Biosharp, Cat. No. BL539A) for 15 min and stained with crystal violet staining solution (Beyotime, Cat. No. C0121-100 ml) for 30 min.

### Determination of half-maximal inhibitory concentration (IC50) synergy

Each well of a 96-well plate was seeded with 500 glioma cells. Cells were treated with PRMT5 and MAT2A inhibitors at concentrations ranging from 1–1000 nM. After 8 days of treatment, the medium was aspirated and replaced with 100 mL of the medium and 10 μL of CCK-8 reagent. The plate was incubated at 37 °C for 3 h for optimal color development of the CCK-8 reagent, and the absorbance was determined at 450 nm. Calibrated data were analyzed for synergy using SynergyFinder 3.0 [37].

### Determination of the cell cycle and apoptosis

Cells were seeded in 6-well plates at a density of 2000 cells per well and treated with the PRMT5 inhibitor and/or MAT2A inhibitor for 8 days. Floating and adherent cells were collected separately and apoptosis of cells was determined using an Annexin V-FITC and propidium iodide (PI) staining kit (BD Biosciences, Cat. No. 556547, 550825) according to the manufacturers’ protocol. Cell cycle changes were assessed by fixing cells in cold ethanol for 1 h and staining with the PI cell cycle kit (Elabscience, Cat. No. E-CK-A351) according to the manufacturers’ protocol. Cells were analyzed using a CytoFlex flow cytometer (Beckman), and data were analyzed using FlowJo V10.8.1, ModFit 5.0.9, and GraphPad Prism 10.3.1.

### Western blotting

Proteins were extracted from cells using IP lysis buffer (Beyotime, Cat. No. P0013). Cell lysates were centrifuged at 12,000 × *g* for 15 min at 4 °C, and the protein concentration of the resulting supernatant was determined using a bicinchoninic acid assay kit (Beyotime, Cat. No. P0010). The extracted proteins were separated using electrophoresis and transferred onto a polyvinylidene fluoride membrane according to their molecular weights. The membrane was blocked with 5% bovine serum albumin (BSA) at room temperature (23 °C) for 1 h, washed 3 times with phosphate-buffered saline with Tween (PBST), and incubated overnight with the primary antibodies at 4 °C. The following day, the membrane was washed 3 times with PBST and incubated with a peroxidase-conjugated secondary antibody at 23 °C for 1 h. Enhanced chemiluminescence reagent (Beyotime, Cat. No. P0018AM) was used to visualize the proteins, and ImageJ software was used to analyze the grayscale values.

### Immunofluorescence

Cells were plated onto slides and fixed with 4% paraformaldehyde for 15 min after 8 days of treatment. After 3 washes with PBST, the cells were fixed in 4% paraformaldehyde for 14 min and permeabilized with 0.3% Triton X-100 for 15 min. Next, the cells were blocked with 5% BSA for 30 min at 23 °C. They were then incubated overnight with the primary antibodies at 4 °C and subsequently incubated with the secondary antibodies for 1 h at 23 °C. Lastly, the coverslips were mounted onto slides using DAPI (SouthernBiotech, Cat. No. 0100-20). Images were acquired using fluorescence microscopy (Keyence).

### Immunohistochemistry

Tissues were fixed in 4% paraformaldehyde, embedded in paraffin, and sectioned using a microtome (LECIA). The sections were deparaffinized at 60 °C for 1 h and then washed with xylene and ethanol. Next, thermal antigen retrieval and quenching of endogenous peroxidase were performed using hydrogen peroxide. Rehydrated and quenched slides were blocked with 10% normal goat serum for 1 h at room temperature. The slides were incubated overnight with the primary antibody (1:400 dilution) at 4 °C. Next, the slides were rinsed 3 times with 1× phosphate-buffered saline (PBS) for 5 min each and incubated with the secondary antibody (1:100) for 1 h at room temperature. The sections were then rinsed 3 times in 1 × PBS for 5 min each and treated with the chromogenic DAB substrate (incubation for 10–50 s) until a sufficient brown color developed. The slides were then washed with 1 × PBS, counterstained with hematoxylin, dehydrated, and mounted for analysis.

### Comet assay

Comet assay was performed to determine DNA damage by using a DNA damage–detection kit (Beyotime, Cat. No. C2041S). Briefly, cells were mixed with low-melting agarose on precoated 2-well comet slides. Cells were then treated with pre-chilled lysis buffer and alkaline solution for 60 and 30 min, respectively, at 4 °C and away from light. This preparation step was performed to facilitate subsequent electrophoresis, and it lasted 10 min. After staining with PI from the comet assay kit, the slides were observed using fluorescence microscopy (Keyence). Tail moment and tail DNA were used to determine DNA damage.

### RNA sequencing and analysis

Total RNA was isolated using Trizol reagent (Thermo Fisher Scientific, Cat. No. 15596018CN) and RNA quality was evaluated. After extraction, eukaryotic cell mRNA was enriched using Oligo(dT) beads. The enriched mRNA was broken down into smaller fragments and used for reverse transcription to synthesize cDNA. The cDNA fragments were ligated using Illumina sequencing adapters. Fragment sizes were selected using agarose gel electrophoresis and amplified using polymerase chain reaction. The resulting cDNA library was sequenced using an Illumina Novaseq6000 platform provided by Gene Denovo Biotechnology Co. (Guangzhou, China). Differential expression and pathway analyses were conducted using Gene Ontology (GO) and Kyoto Encyclopedia of Genes and Genomes (KEGG) analyses.

### Animal experiments

Male BALB/c nude mice (6–8 weeks old) were purchased from the Institute of Oncology, Chinese Academy of Medical Sciences (Beijing, China) and raised in a pathogen-free environment. We randomly divided the experimental animals into groups for subsequent experiments. A total of 3e5 U87 cells with luciferase-encoded lentivirus (Gene Chem, China) were injected into the frontal subdural region of mice. Intracranial tumor growth was recorded using an IVIS imaging system (Caliper Life Sciences) after injecting with D-luciferin (Solarbio, China) on days 0, 7, 14, and 21. Mice were euthanized on day 35, and their brains were collected and fixed using 4% paraformaldehyde. All samples were embedded in paraffin and sliced for hematoxylin and eosin (H&E) staining and immunohistochemical assay. All animal studies were conducted in accordance with the Guidelines of the Ethics Committee of Soochow University.

### Statistical analysis

All experiments were performed at least 3 times, and data are expressed as the mean ± standard deviation (SD). Statistical analyses were performed using GraphPad Prism 10. Differences between groups were assessed using the log-rank test, *t*-test, and one-way analysis of variance. *P* < 0.05 was considered statistically significant.

## Supplementary information

Supplementary Figure legends

Supplementary Figure 1

Supplementary Figure 2

Supplementary Figure 3

Original Data

## Data Availability

The data that support the findings of this study are available on request from the corresponding author. The data are not publicly available due to privacy or ethical restrictions.

## References

[1] Ostrom QT, Gittleman H, Liao P, Rouse C, Chen Y, Dowling J, et al. CBTRUS statistical report: primary brain and central nervous system tumors diagnosed in the United States in 2007-2011. Neuro Oncol. 2014;16:iv1–63. 25304271 10.1093/neuonc/nou223 PMC4193675

[2] Weller M, van den Bent M, Preusser M, Le Rhun E, Tonn JC, Minniti G, et al. Author Correction: EANO guidelines on the diagnosis and treatment of diffuse gliomas of adulthood. Nat Rev Clin Oncol. 2022;19:357–8. 35322237 10.1038/s41571-022-00623-3 PMC9038523

[3] Wick W, Gorlia T, Bendszus M, Taphoorn M, Sahm F, Harting I, et al. Lomustine and bevacizumab in progressive glioblastoma. N Engl J Med. 2017;377:1954–63. 29141164 10.1056/NEJMoa1707358

[4] Lucchesi JC. Synthetic lethality and semi-lethality among functionally related mutants of Drosophila melanfgaster. Genetics. 1968;59:37–44. 5683639 10.1093/genetics/59.1.37 PMC1211931

[5] Farmer H, McCabe N, Lord CJ, Tutt ANJ, Johnson DA, Richardson TB, et al. Targeting the DNA repair defect in BRCA mutant cells as a therapeutic strategy. Nature. 2005;434:917–21. 15829967 10.1038/nature03445

[6] Parsons DW, Jones S, Zhang X, Lin JC-H, Leary RJ, Angenendt P, et al. An integrated genomic analysis of human glioblastoma multiforme. Science. 2008;321:1807–12. 18772396 10.1126/science.1164382 PMC2820389

[7] Antonysamy S. The structure and function of the PRMT5:MEP50 complex. Subcell Biochem. 2017;83:185–94. 28271477 10.1007/978-3-319-46503-6_7

[8] Sun L, Wang M, Lv Z, Yang N, Liu Y, Bao S, et al. Structural insights into protein arginine symmetric dimethylation by PRMT5. Proc Natl Acad Sci USA. 2011;108:20538–43. 22143770 10.1073/pnas.1106946108 PMC3251124

[9] Gu Z, Gao S, Zhang F, Wang Z, Ma W, Davis RE, et al. Protein arginine methyltransferase 5 is essential for growth of lung cancer cells. Biochem J. 2012;446:235–41. 22708516 10.1042/BJ20120768 PMC3865921

[10] Yan F, Alinari L, Lustberg ME, Martin LK, Cordero-Nieves HM, Banasavadi-Siddegowda Y, et al. Genetic validation of the protein arginine methyltransferase PRMT5 as a candidate therapeutic target in glioblastoma. Cancer Res. 2014;74:1752–65. 24453002 10.1158/0008-5472.CAN-13-0884 PMC3959215

[11] Banasavadi-Siddegowda YK, Welker AM, An M, Yang X, Zhou W, Shi G, et al. PRMT5 as a druggable target for glioblastoma therapy. Neuro Oncol. 2018;20:753–63. 29106602 10.1093/neuonc/nox206 PMC5961180

[12] Banasavadi-Siddegowda YK, Russell L, Frair E, Karkhanis VA, Relation T, Yoo JY, et al. PRMT5-PTEN molecular pathway regulates senescence and self-renewal of primary glioblastoma neurosphere cells. Oncogene. 2017;36:263–74. 27292259 10.1038/onc.2016.199 PMC5240810

[13] Braun CJ, Stanciu M, Boutz PL, Patterson JC, Calligaris D, Higuchi F, et al. Coordinated splicing of regulatory detained introns within oncogenic transcripts creates an exploitable vulnerability in malignant glioma. Cancer Cell. 2017;32:411–426.e11. 28966034 10.1016/j.ccell.2017.08.018 PMC5929990

[14] Du C, Hansen LJ, Singh SX, Wang F, Sun R, Moure CJ, et al. A PRMT5-RNF168-SMURF2 axis controls H2AX proteostasis. Cell Rep. 2019;28:3199–3211.e5. 31533041 10.1016/j.celrep.2019.08.031 PMC7204040

[15] Gerhart SV, Kellner WA, Thompson C, Pappalardi MB, Zhang X-P, Montes de Oca R, et al. Activation of the p53-MDM4 regulatory axis defines the anti-tumour response to PRMT5 inhibition through its role in regulating cellular splicing. Sci Rep. 2018;8:9711. 29946150 10.1038/s41598-018-28002-y PMC6018746

[16] Marjon K, Cameron MJ, Quang P, Clasquin MF, Mandley E, Kunii K, et al. MTAP deletions in cancer create vulnerability to targeting of the MAT2A/PRMT5/RIOK1 axis. Cell Rep. 2016;15:574–87. 27068473 10.1016/j.celrep.2016.03.043

[17] Kryukov GV, Wilson FH, Ruth JR, Paulk J, Tsherniak A, Marlow SE, et al. MTAP deletion confers enhanced dependency on the PRMT5 arginine methyltransferase in cancer cells. Science. 2016;351:1214–8. 26912360 10.1126/science.aad5214 PMC4997612

[18] Mavrakis KJ, McDonald ER 3rd, Schlabach MR, Billy E, Hoffman GR, deWeck A, et al. Disordered methionine metabolism in MTAP/CDKN2A-deleted cancers leads to dependence on PRMT5. Science. 2016;351:1208–13. 26912361 10.1126/science.aad5944

[19] Lu SC, Mato JM. S-adenosylmethionine in liver health, injury, and cancer. Physiol Rev. 2012;92:1515–42. 23073625 10.1152/physrev.00047.2011 PMC3698976

[20] Murray B, Antonyuk SV, Marina A, Lu SC, Mato JM, Hasnain SS, et al. Crystallography captures catalytic steps in human methionine adenosyltransferase enzymes. Proc Natl Acad Sci USA. 2016;113:2104–9. 26858410 10.1073/pnas.1510959113 PMC4776477

[21] Cai J, Mao Z, Hwang JJ, Lu SC. Differential expression of methionine adenosyltransferase genes influences the rate of growth of human hepatocellular carcinoma cells. Cancer Res. 1998;58:1444–50. 9537246

[22] Yang H, Huang ZZ, Wang J, Lu SC. The role of c-Myb and Sp1 in the up-regulation of methionine adenosyltransferase 2A gene expression in human hepatocellular carcinoma. FASEB J. 2001;15:1507–16. 11427482 10.1096/fj.01-0040com

[23] Yang H, Sadda MR, Yu V, Zeng Y, Lee TD, Ou X, et al. Induction of human methionine adenosyltransferase 2A expression by tumor necrosis factor alpha. Role of NF-kappa B and AP-1. J Biol Chem. 2003;278:50887–96. 14530285 10.1074/jbc.M307600200

[24] Ito K, Ikeda S, Kojima N, Miura M, Shimizu-Saito K, Yamaguchi I, et al. Correlation between the expression of methionine adenosyltransferase and the stages of human colorectal carcinoma. Surg Today. 2000;30:706–10. 10955733 10.1007/s005950070081

[25] Chen H, Xia M, Lin M, Yang H, Kuhlenkamp J, Li T, et al. Role of methionine adenosyltransferase 2A and S-adenosylmethionine in mitogen-induced growth of human colon cancer cells. Gastroenterology. 2007;133:207–18. 17631143 10.1053/j.gastro.2007.03.114

[26] Faridoon, Zheng J, Zhang T, Tong S, Liu T, Cui J, et al. Structure-based design and optimization of methionine adenosyltransferase 2A (MAT2A) inhibitors with high selectivity, brain penetration, and in vivo efficacy. J Med Chem. 2024;67:9431–46. 38818879 10.1021/acs.jmedchem.4c00552

[27] Krause K, Wasner M, Reinhard W, Haugwitz U, Dohna CL, Mössner J, et al. The tumour suppressor protein p53 can repress transcription of cyclin B. Nucleic Acids Res. 2000;28:4410–8. 11071927 10.1093/nar/28.22.4410 PMC113869

[28] Porter LA, Donoghue DJ. Cyclin B1 and CDK1: nuclear localization and upstream regulators. Prog Cell Cycle Res. 2003;5:335–47. 14593728

[29] Belmontes B, Slemmons KK, Su C, Liu S, Policheni AN, Moriguchi J, et al. AMG 193, a clinical stage MTA-cooperative PRMT5 inhibitor, drives antitumor activity preclinically and in patients with MTAP-deleted cancers. Cancer Discov. 2025;15:139–61. 39282709 10.1158/2159-8290.CD-24-0887 PMC11726016

[30] Jo H, Mondal S, Tan D, Nagata E, Takizawa S, Sharma AK, et al. Small molecule-induced cytosolic activation of protein kinase Akt rescues ischemia-elicited neuronal death. Proc Natl Acad Sci USA. 2012;109:10581–6. 22689977 10.1073/pnas.1202810109 PMC3387065

[31] Li X, Wang C, Jiang H, Luo C. A patent review of arginine methyltransferase inhibitors (2010-2018). Expert Opin Ther Pat. 2019;29:97–114. 30640571 10.1080/13543776.2019.1567711

[32] Tao H, Yan X, Zhu K, Zhang H. Discovery of Novel PRMT5 Inhibitors by Virtual Screening and Biological Evaluations. Chem Pharm Bull. 2019;67:382–8. 10.1248/cpb.c18-00980 30930442

[33] Chan-Penebre E, Kuplast KG, Majer CR, Boriack-Sjodin PA, Wigle TJ, Johnston LD, et al. A selective inhibitor of PRMT5 with in vivo and in vitro potency in MCL models. Nat Chem Biol. 2015;11:432–7. 25915199 10.1038/nchembio.1810

[34] Otani Y, Sur HP, Rachaiah G, Namagiri S, Chowdhury A, Lewis CT, et al. Inhibiting protein phosphatase 2A increases the antitumor effect of protein arginine methyltransferase 5 inhibition in models of glioblastoma. Neuro Oncol. 2021;23:1481–93. 33556161 10.1093/neuonc/noab014 PMC8408848

[35] Zhang Y, Shao Y, Li Y, Li X, Zhang X, EQ, et al. The generation of glioma organoids and the comparison of two culture methods. Cancer Med. 2024;13:e7081. 38457217 10.1002/cam4.7081 PMC10923046

[36] Cancer Genome Atlas Research Network, Weinstein JN, Collisson EA, Mills GB, Shaw KRM, Ozenberger BA, et al. The Cancer Genome Atlas Pan-Cancer analysis project. Nat Genet. 2013;45:1113–20. 24071849 10.1038/ng.2764 PMC3919969

[37] Ianevski A, Giri AK, Aittokallio T. SynergyFinder 3.0: an interactive analysis and consensus interpretation of multi-drug synergies across multiple samples. Nucleic Acids Res. 2022;50:W739–43. 35580060 10.1093/nar/gkac382 PMC9252834

