# Supplementary material for: Combined inhibition by PRMT5 and MAT2A demonstrates a strong synthetic lethality in MTAP homozygous-deficient glioma models
Source: Cell Death Discov. 2025 May 31;11:261. doi: 10.1038/s41420-025-02545-2 (PMC12126582; doi:10.1038/s41420-025-02545-2)
Supplement: Supplementary file 1 — Supplementary Figure legends [file 41420_2025_2545_MOESM1_ESM.doc]

**Supplementary Figure legends:**

**Supplementary Figure 1: Transcriptome sequencing suggesting inhibition of the PI3k/Akt pathway after treatment**

(A) Heatmaps indicating changes in gene expression in the 4 groups (NC, PRMT5i: 10 nM; MAT2Ai: 10 nM; combination: PRMT5i: 10 nM + MAT2Ai: 10 nM). (B) GO enrichment analysis for the 4 groups. (C) KEGG enrichment analysis for the 4 groups. (D) Western blotting to determine changes in protein expression trends of Akt, P-Akt, PI3k, and P-PI3k in these 3 cell lines (PRMT5i: 10 nM; MAT2Ai: 10 nM) and their statistical charts (n=3)

**Supplementary Figure 2: SC79 can partially reverse the cell-killing and proliferation-inhibition effect of the inhibitors**

(A) CCK-8 assays for LN18 and U87 cells (SC79: 10 μM; combination: PRMT5i: 10 nM + MAT2Ai: 10 nM) (B) Colony formation assays for LN18 and U87 cells (SC79: 10 μM; combination: PRMT5i: 10 nM + MAT2Ai: 10 nM), and its statistical charts (n=3). (C) Flow cytometry to determine apoptosis in LN18 and U87 cells (SC79: 10 μM; combination: PRMT5i: 10 nM + MAT2Ai: 10 nM), and its statistical charts (n=3). (D, F) Edu fluorescence levels in LN18 and U87 cells (SC79: 10 μM; combination: PRMT5i: 10 nM + MAT2Ai: 10 nM), and its statistical charts (n=3). (E, G) TUNEL assay indicating fluorescence levels in LN18 and U87 cells (SC79: 10 μM; combination: PRMT5i: 10 nM + MAT2Ai: 10 nM), and its statistical charts (n=3). One-way ANOVA for multi-group comparisons. *p < 0.05, **p < 0.01, ***p < 0.001, ****p < 0.0001.

**Supplementary Figure 3**: DE analysis of the 4 groups (NC, PRMT5i: 10 nM, MAT2Ai: 10 nM; combination: PRMT5i: 10 nM + MAT2Ai: 10 nM)
