# Supplementary figures and images for: Combined inhibition by PRMT5 and MAT2A demonstrates a strong synthetic lethality in MTAP homozygous-deficient glioma models
Source: Cell Death Discov. 2025 May 31;11:261. doi: 10.1038/s41420-025-02545-2 (PMC12126582; doi:10.1038/s41420-025-02545-2)

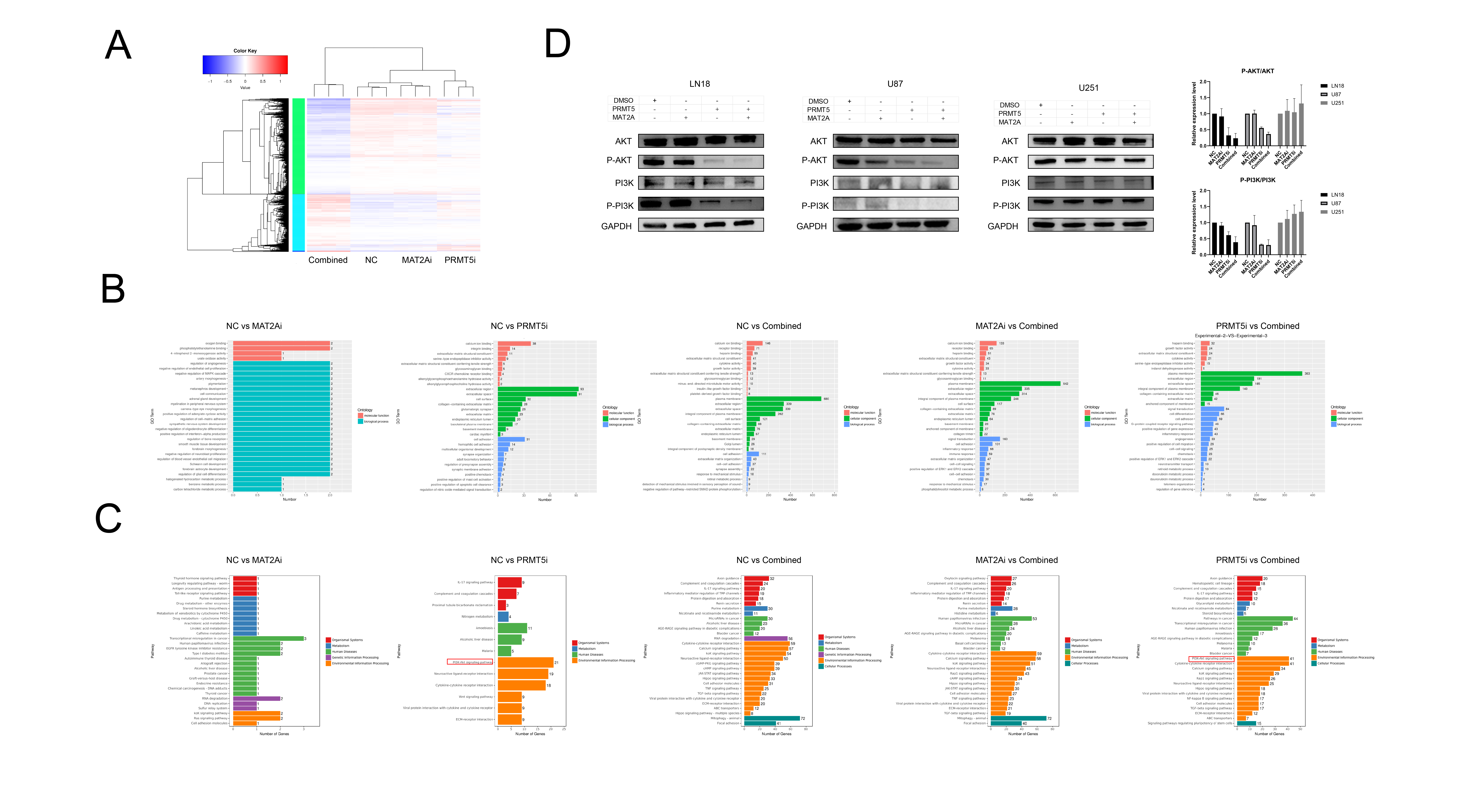

Supplement: Supplementary file 2 — Supplementary Figure 1 [file 41420_2025_2545_MOESM2_ESM.tif]

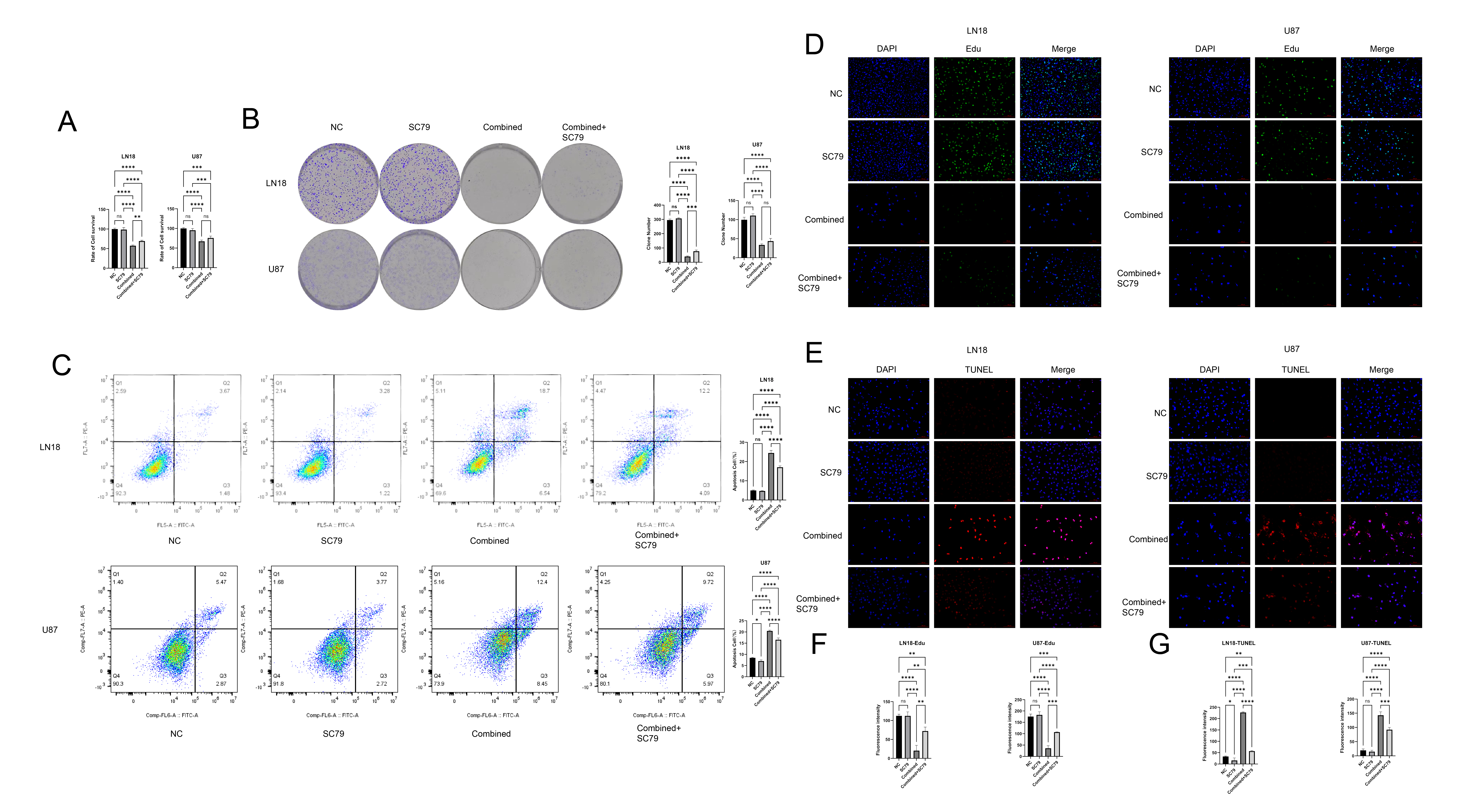

Supplement: Supplementary file 3 — Supplementary Figure 2 [file 41420_2025_2545_MOESM3_ESM.tif]

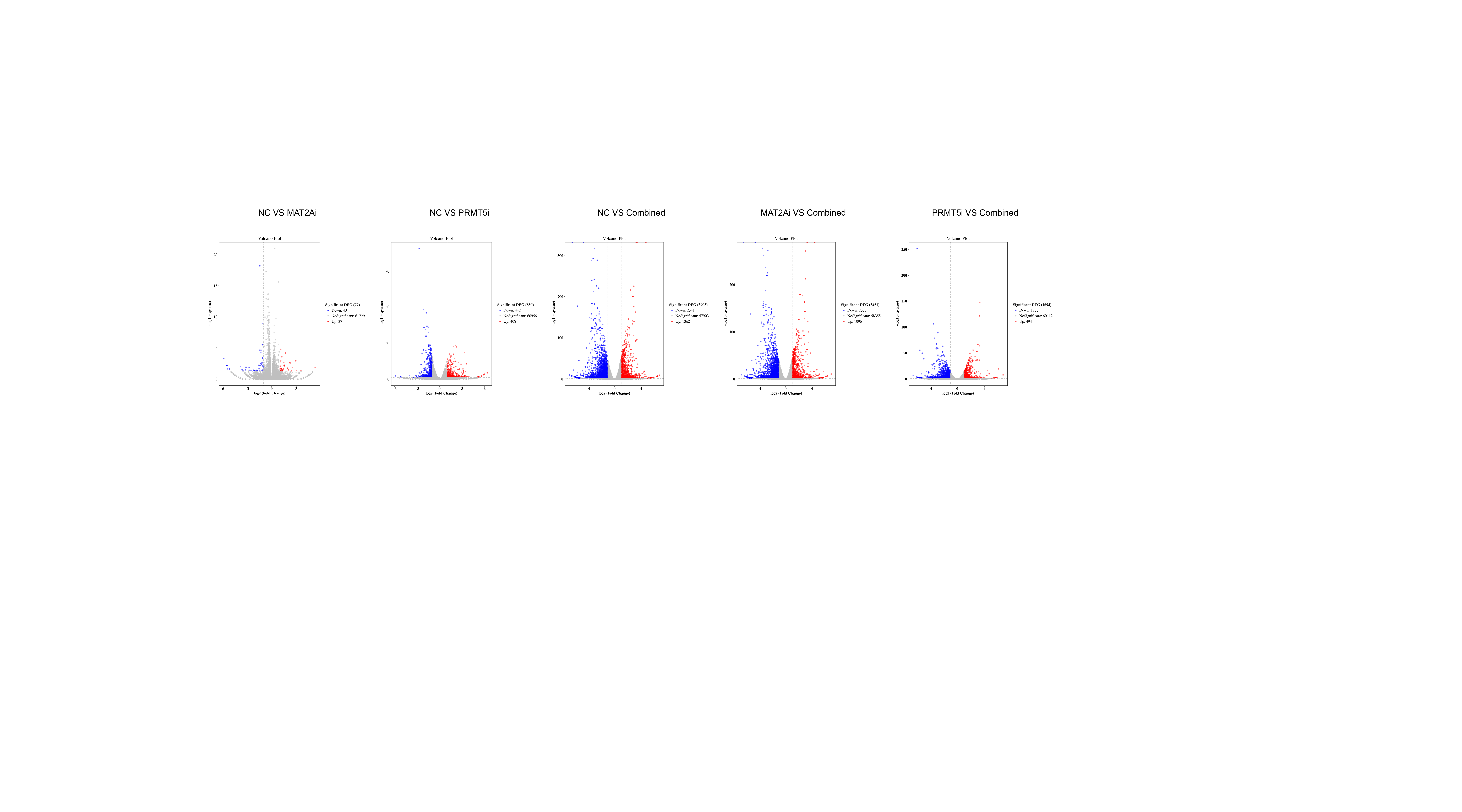

Supplement: Supplementary file 4 — Supplementary Figure 3 [file 41420_2025_2545_MOESM4_ESM.tif]

**Figure1:**


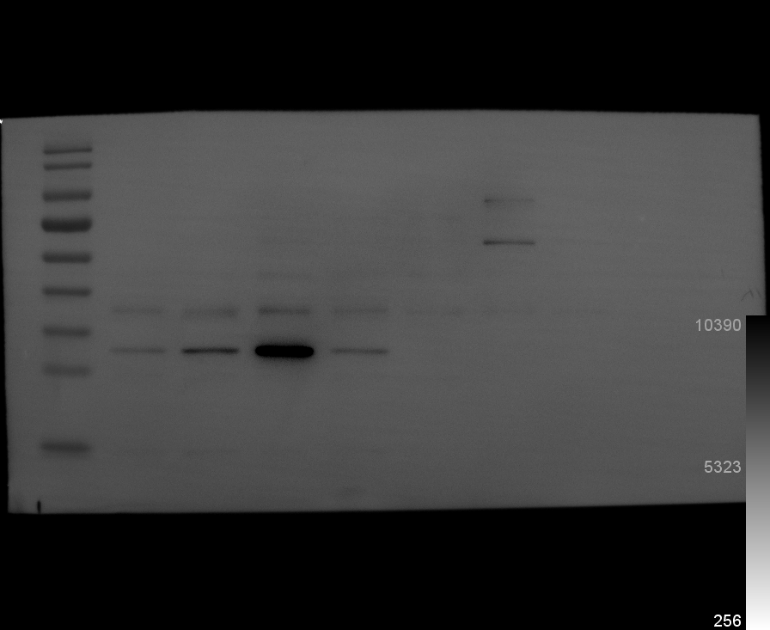

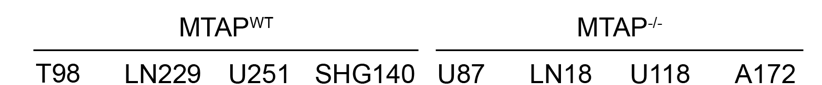

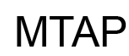

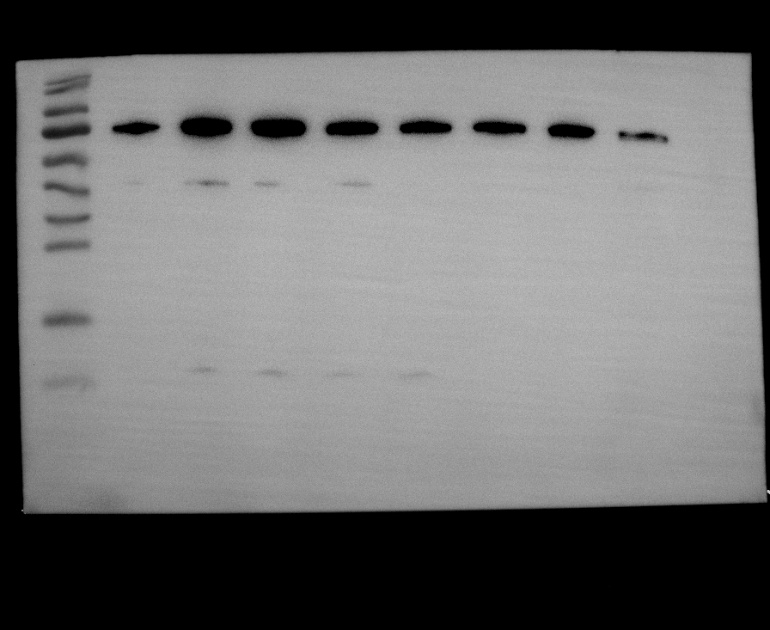

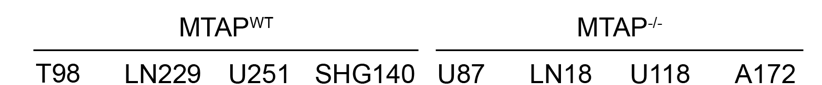

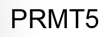

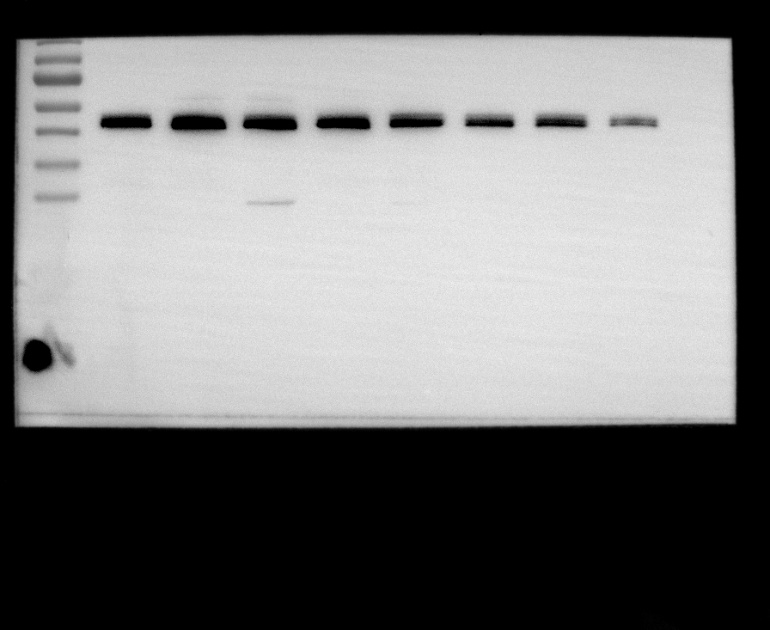

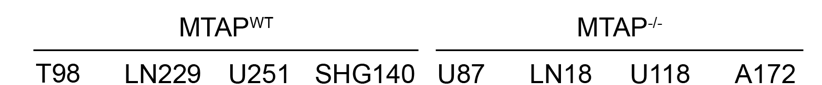

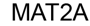

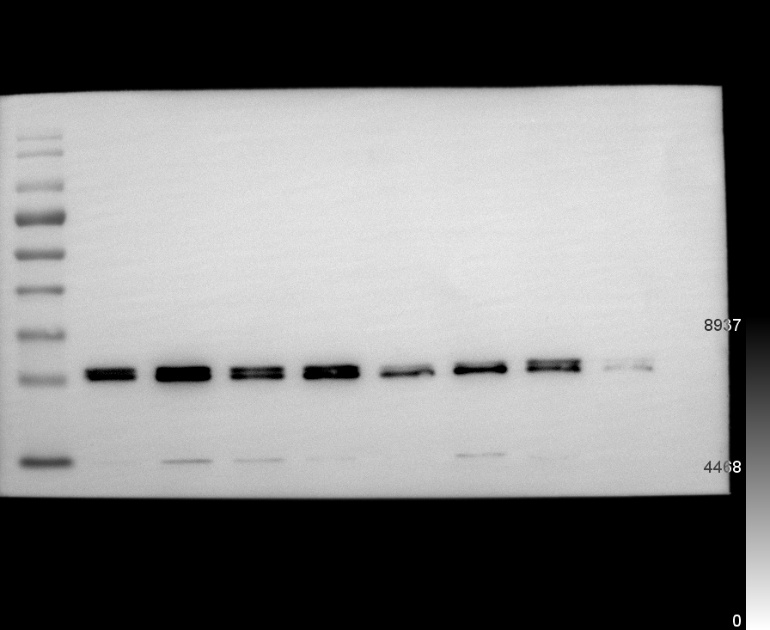

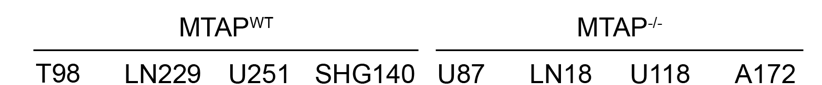

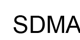

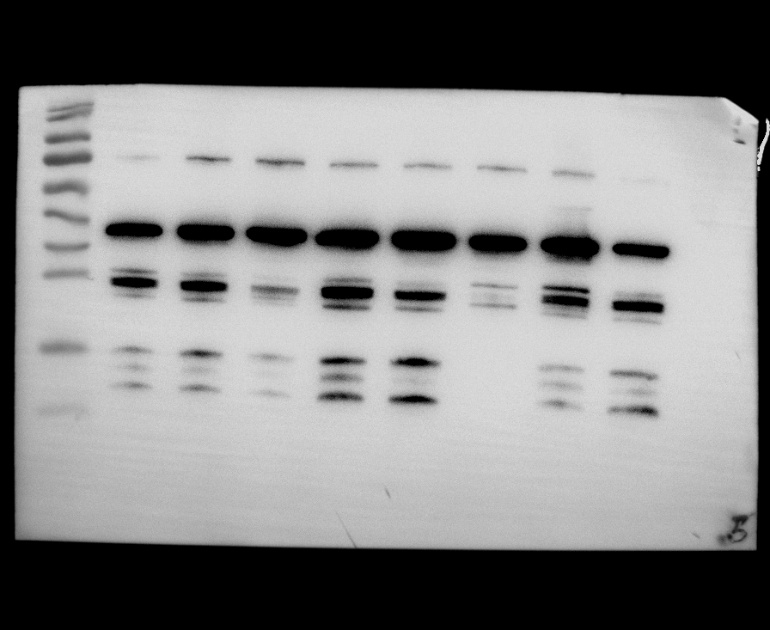

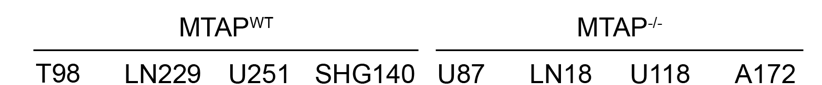

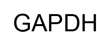


**Figure2:**


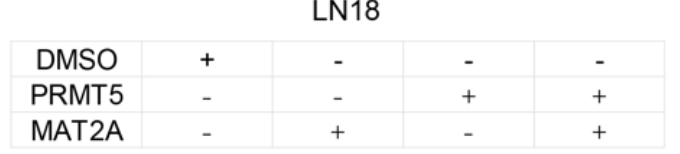


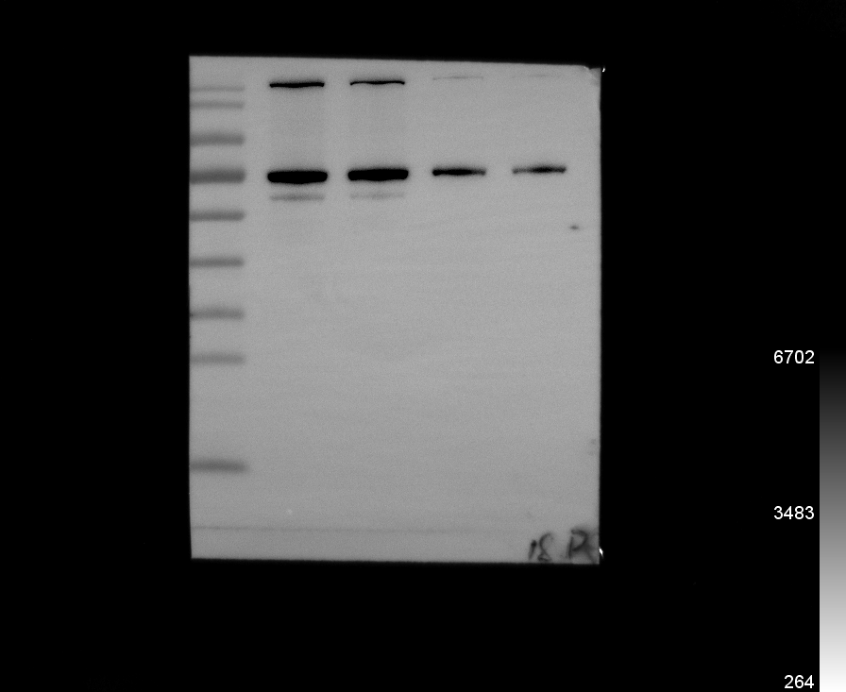


\


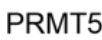


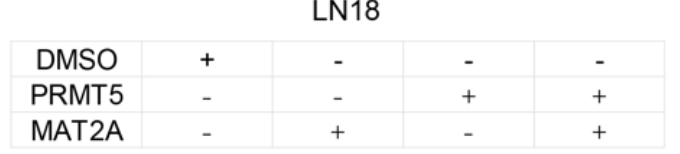

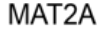

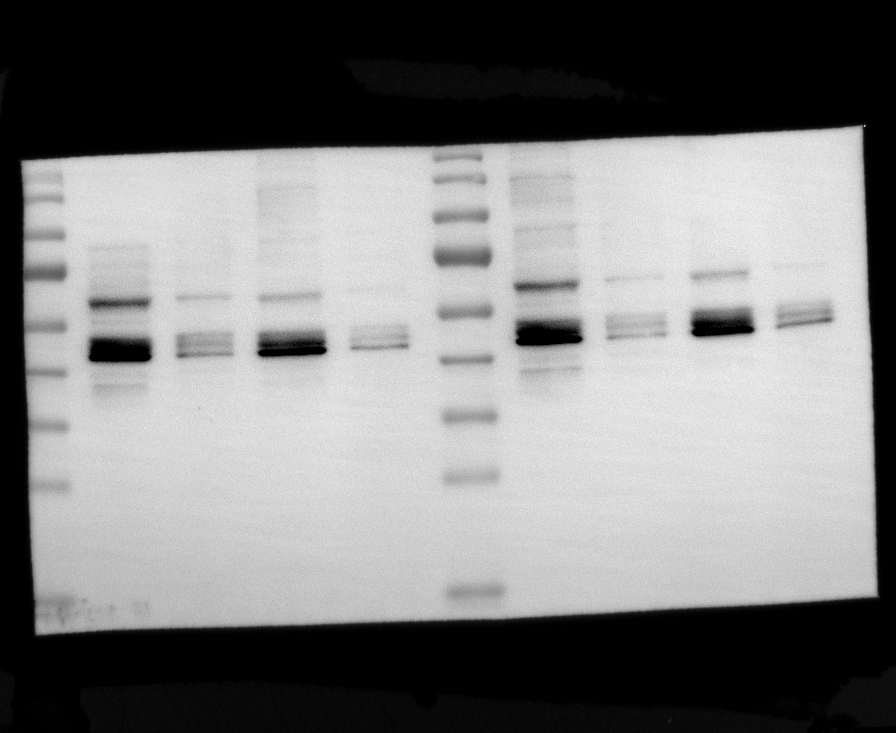


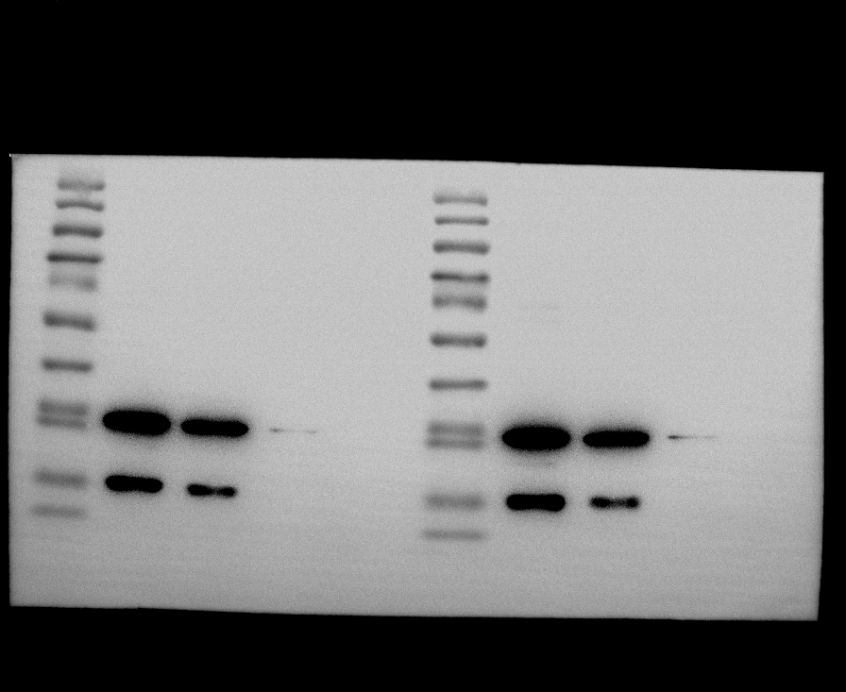


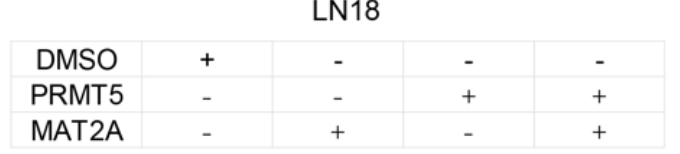


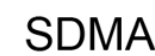


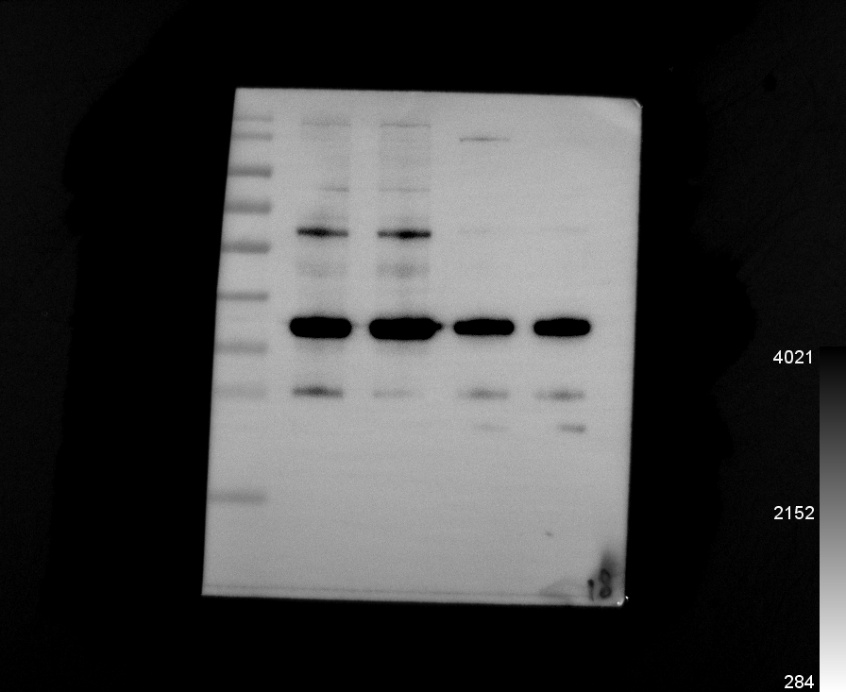

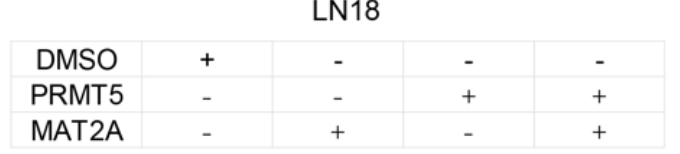


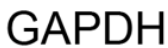


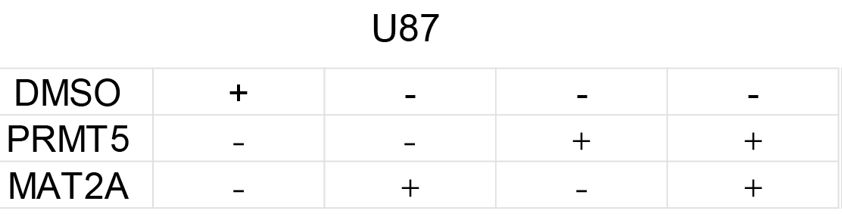


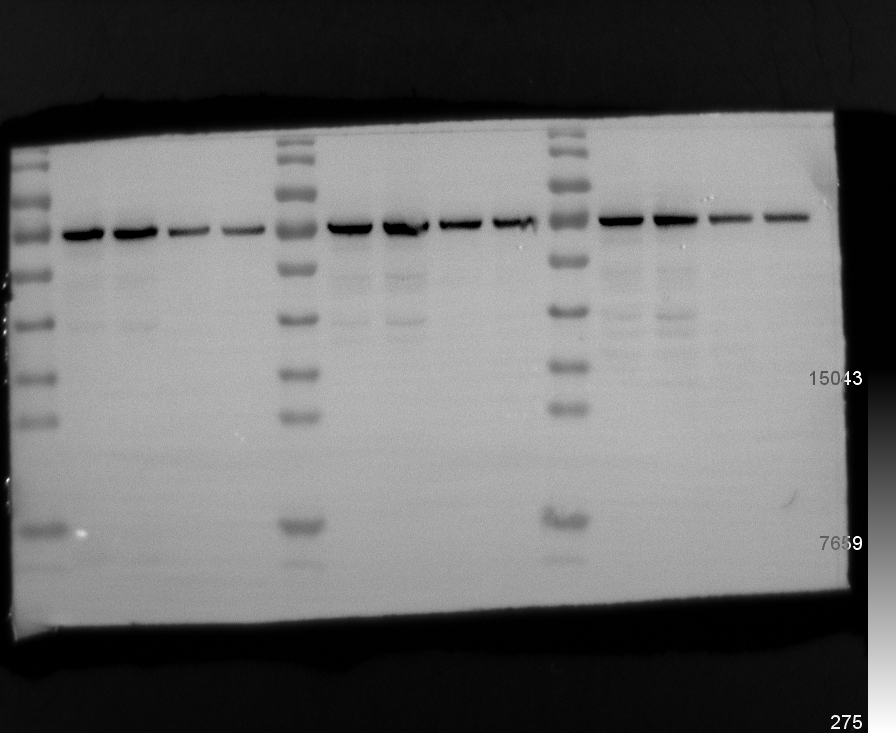


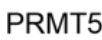


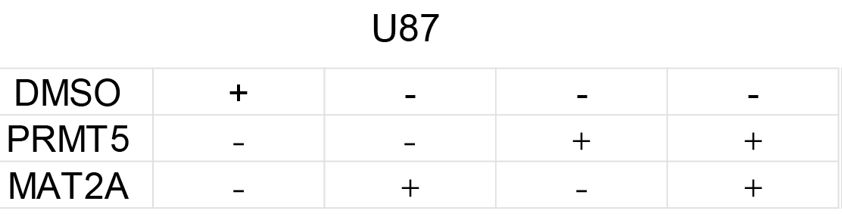


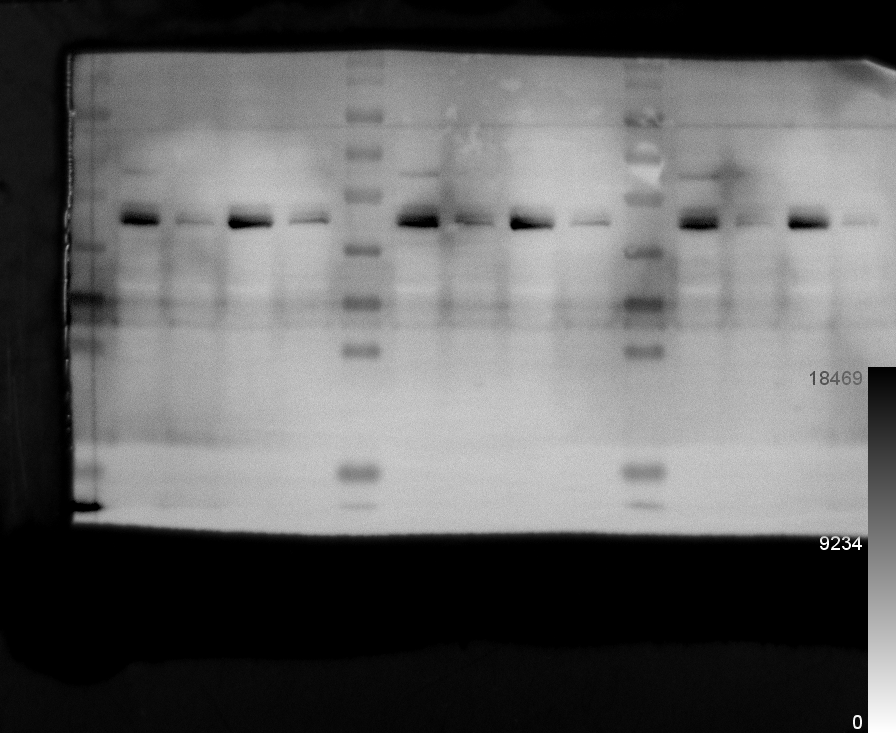


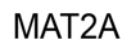


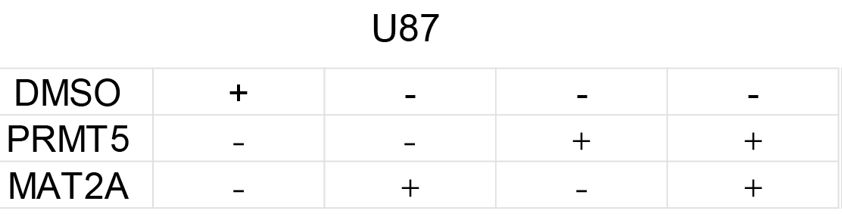


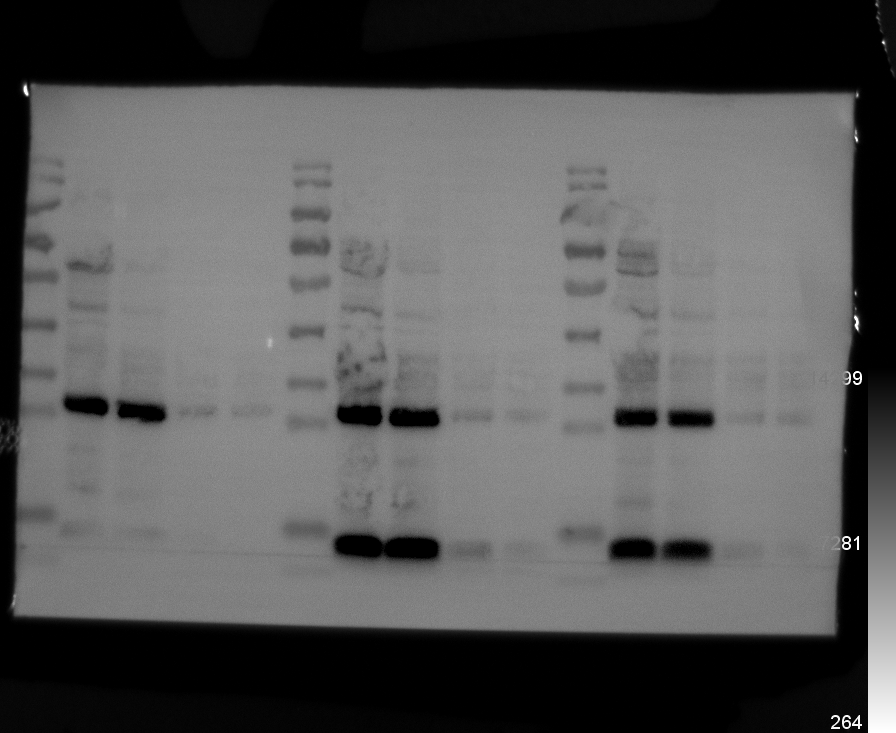


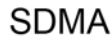


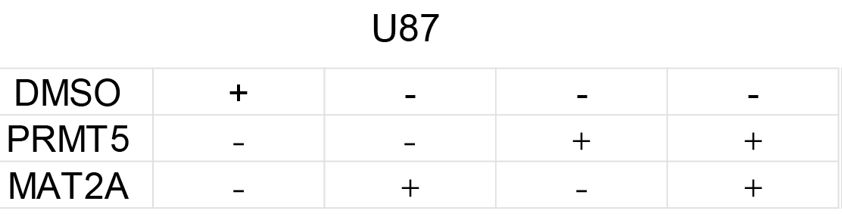


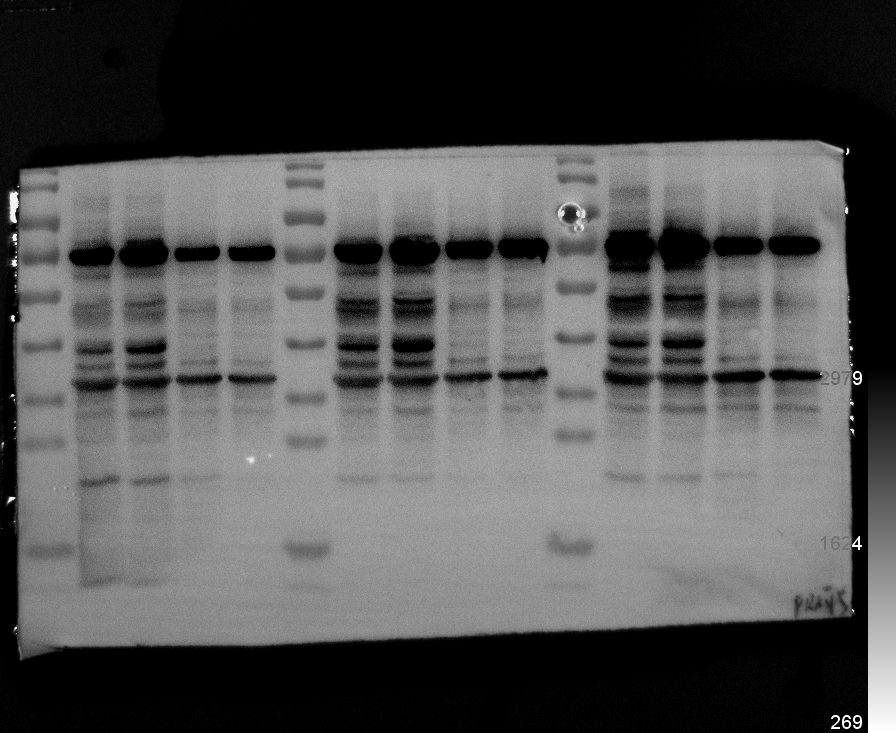


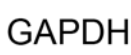


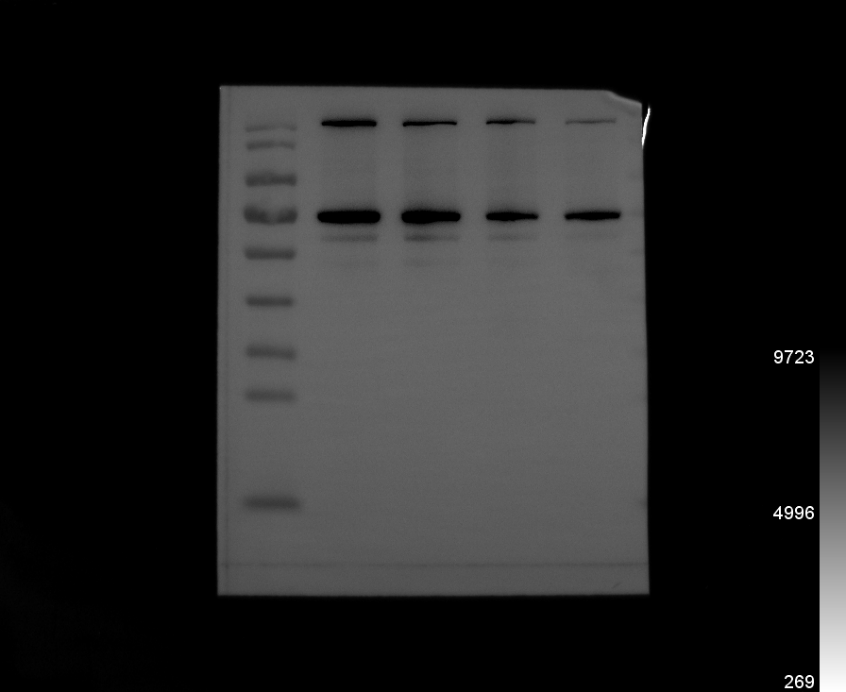

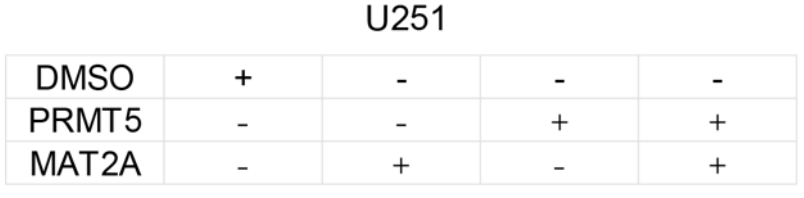


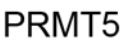


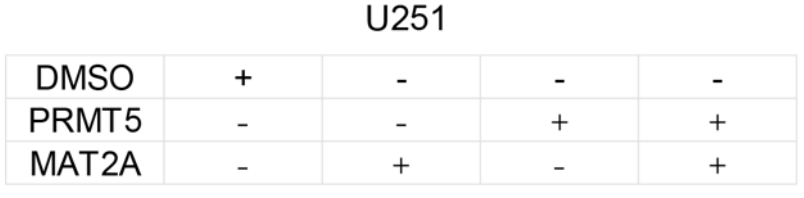


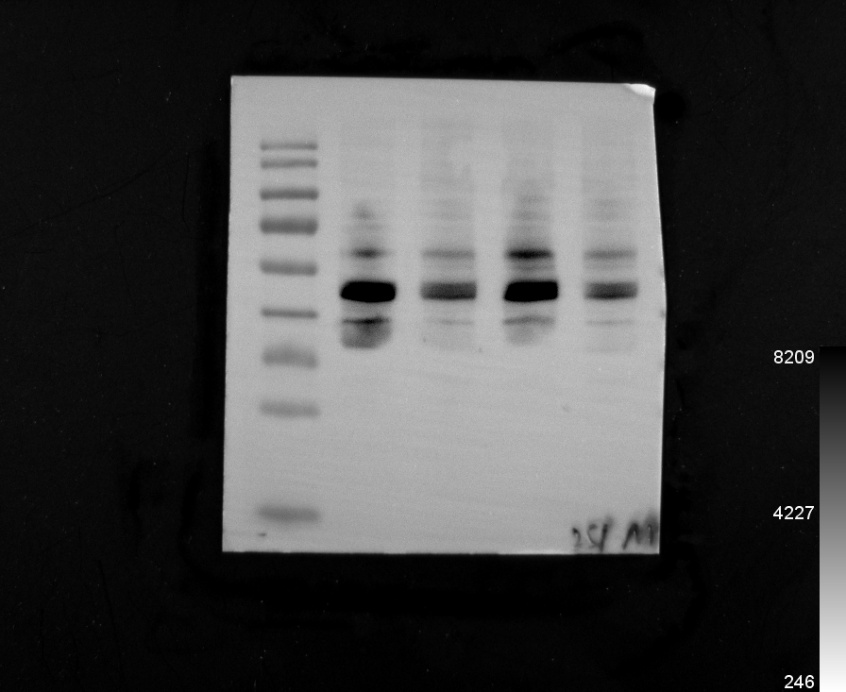
\


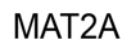


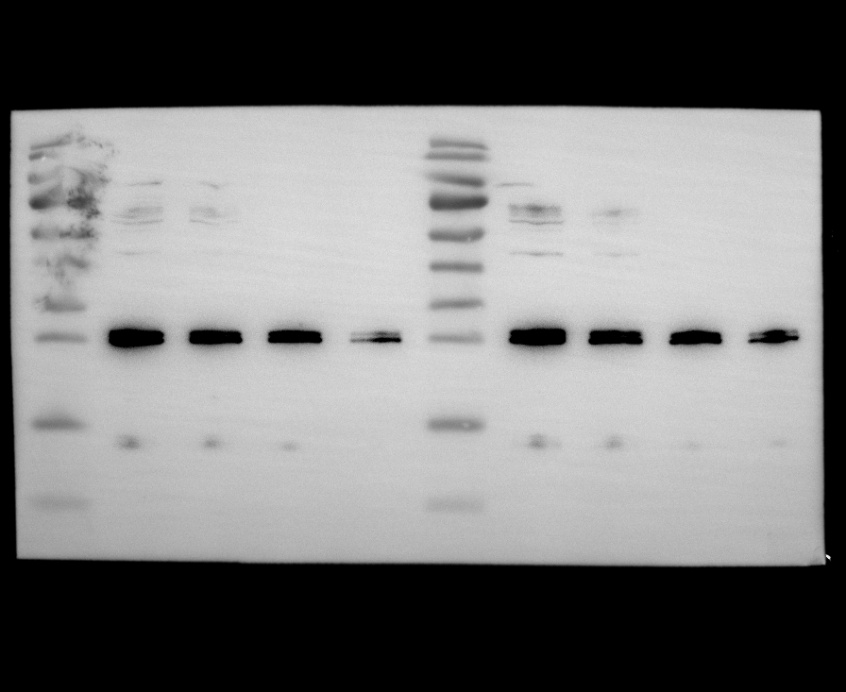

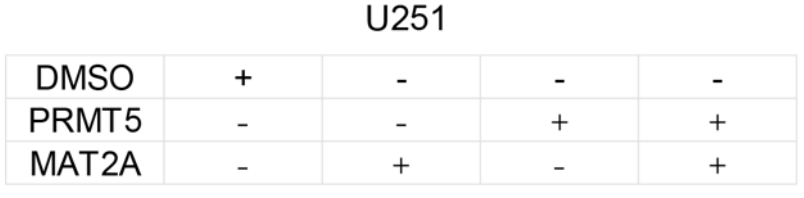


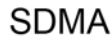


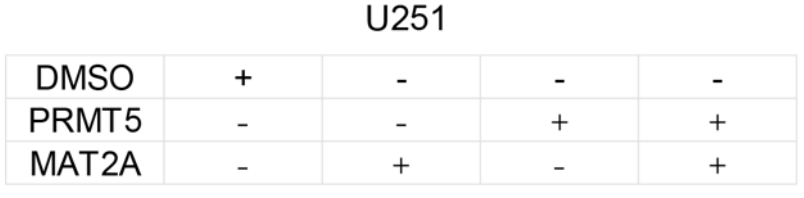


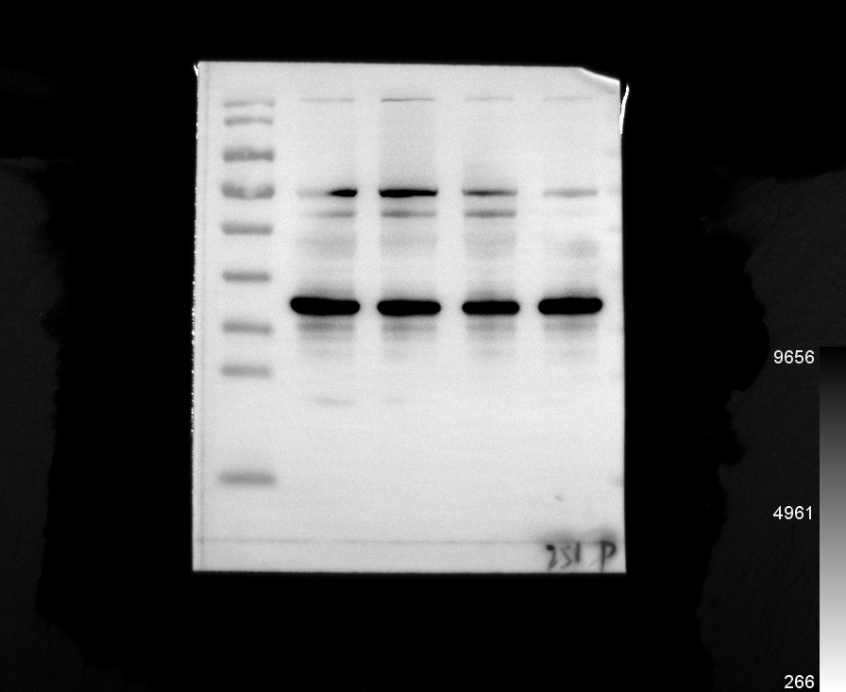


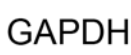


**Figure3:**


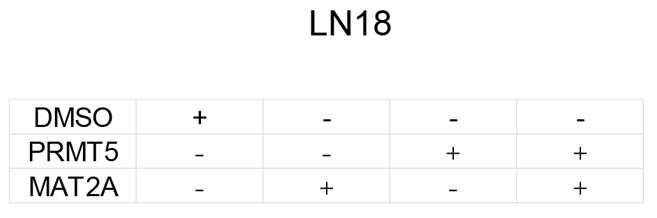


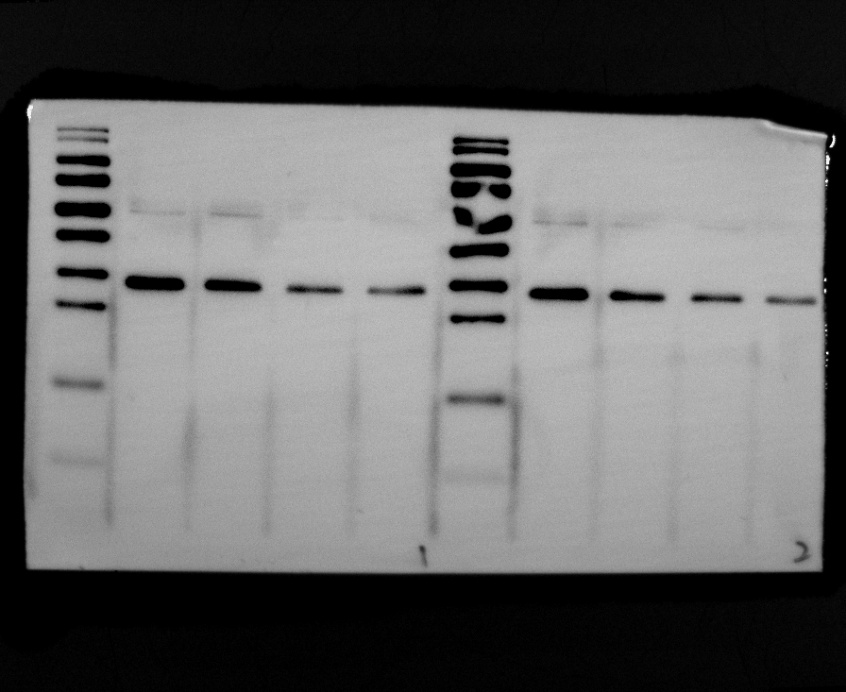


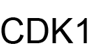


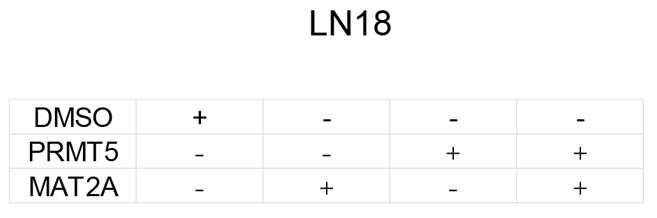


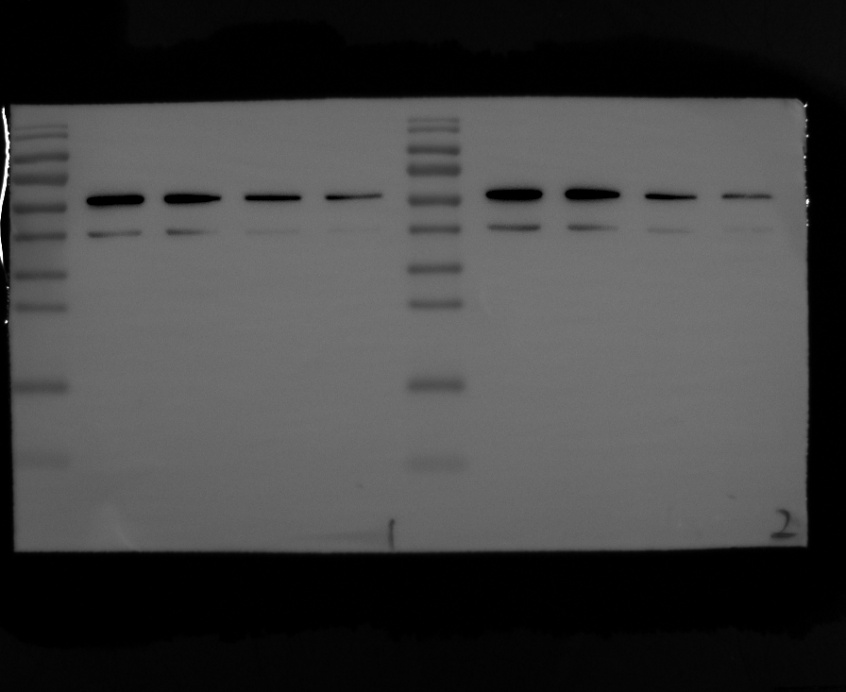


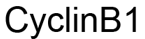


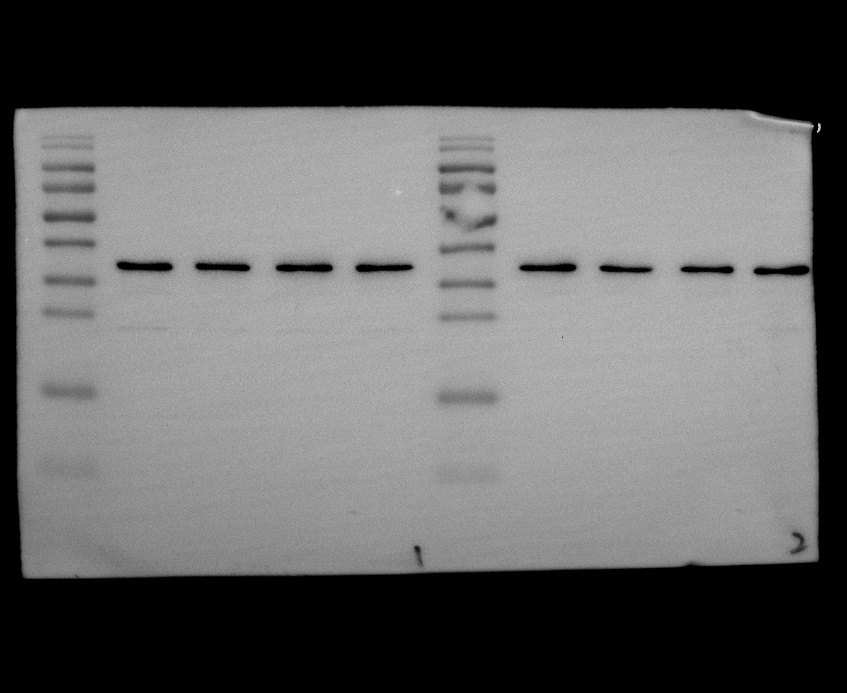

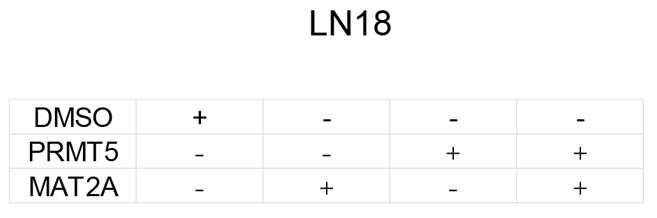

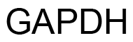


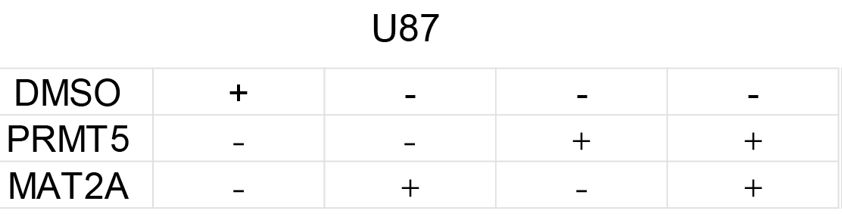


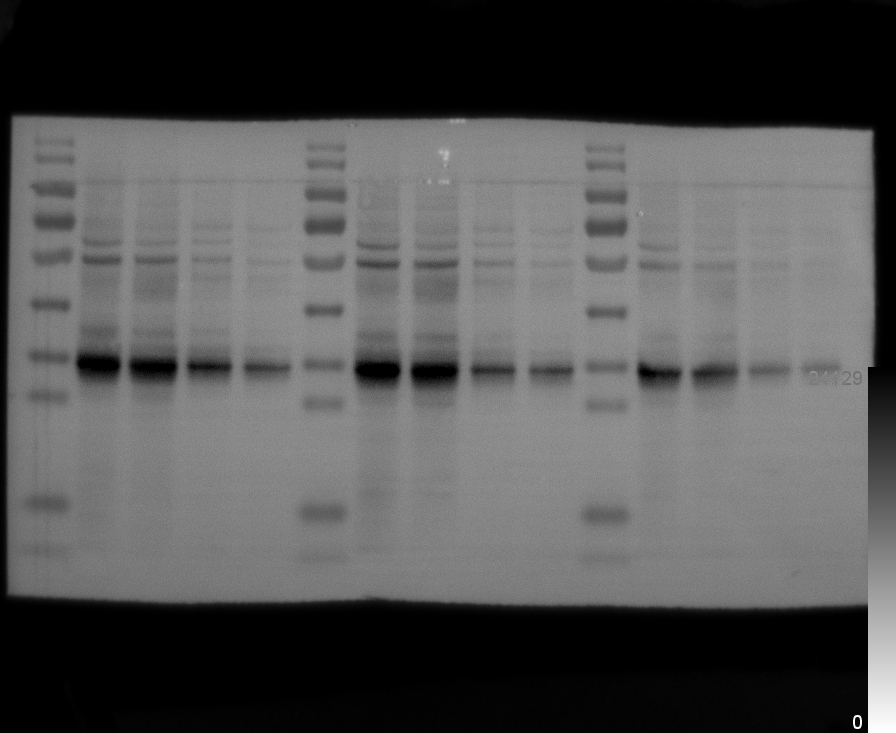


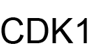


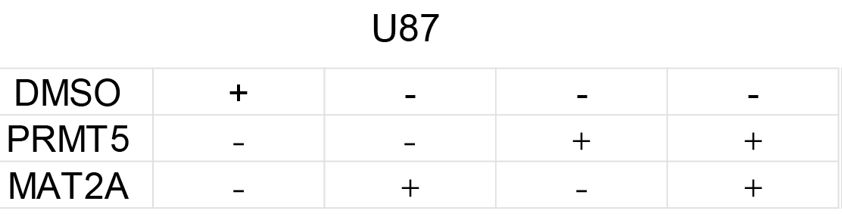


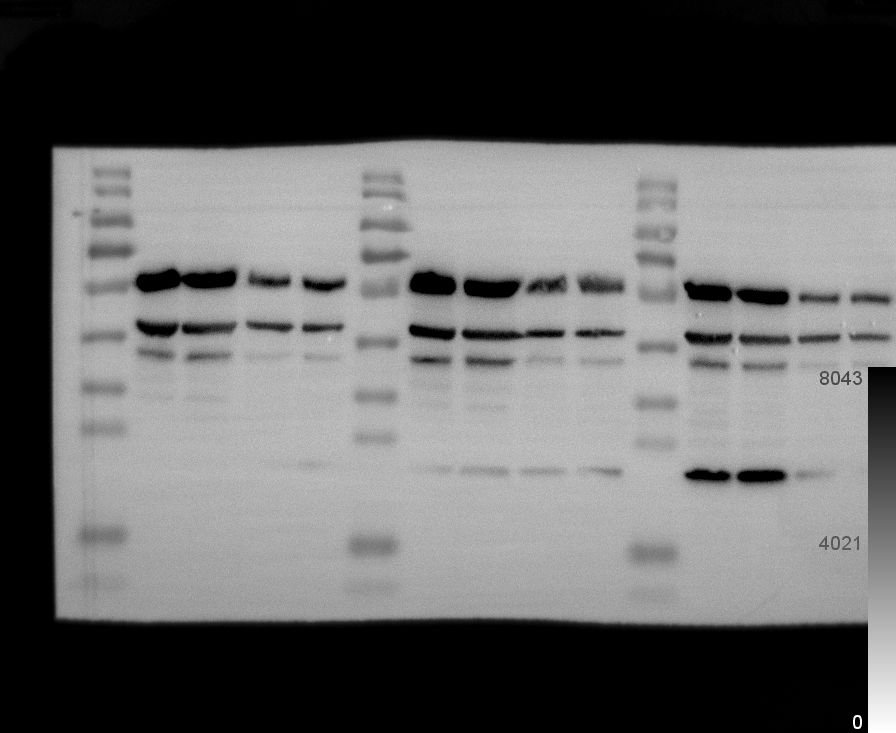


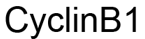


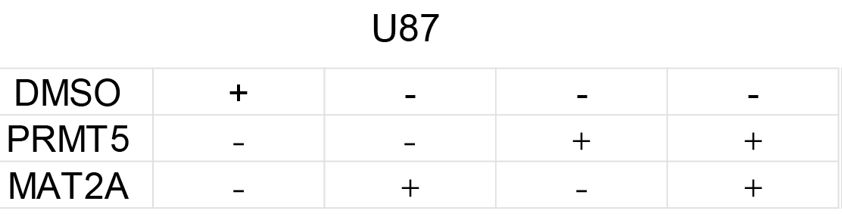


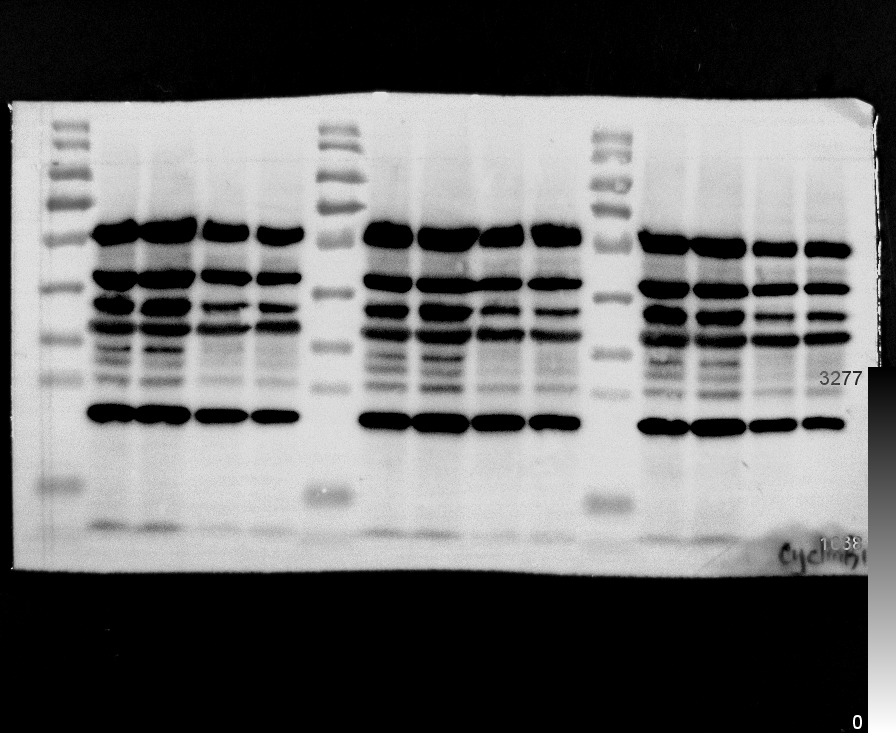


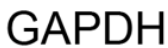


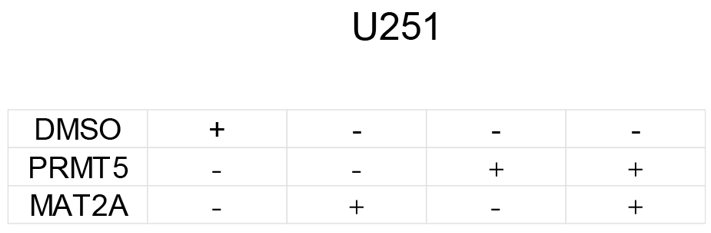


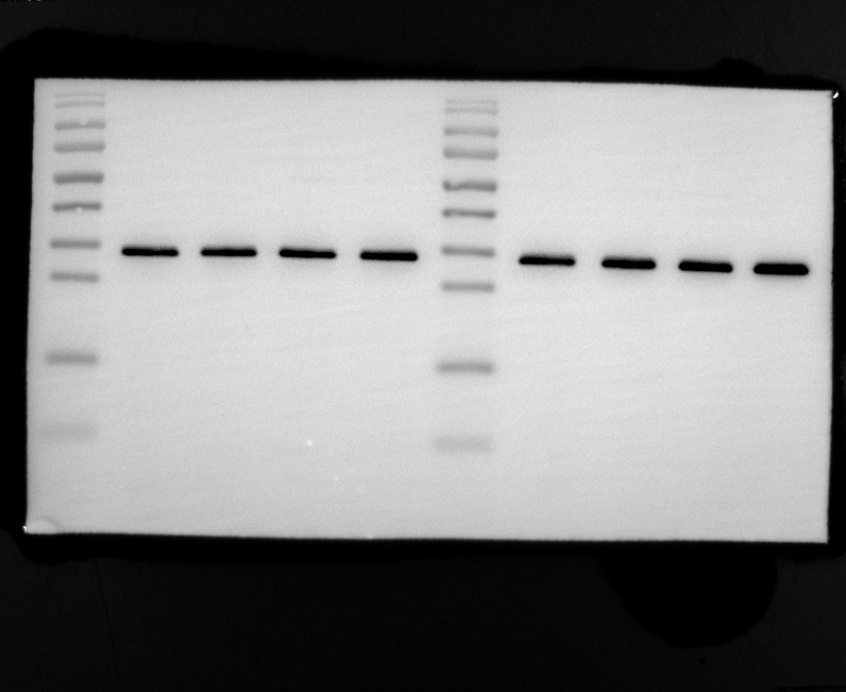


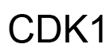


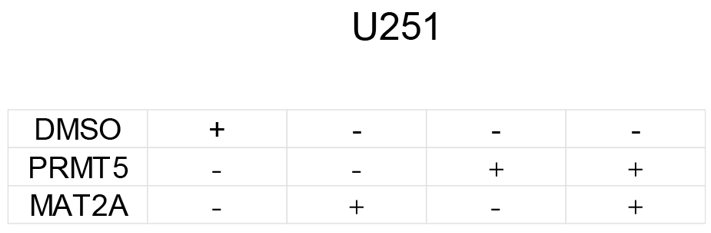


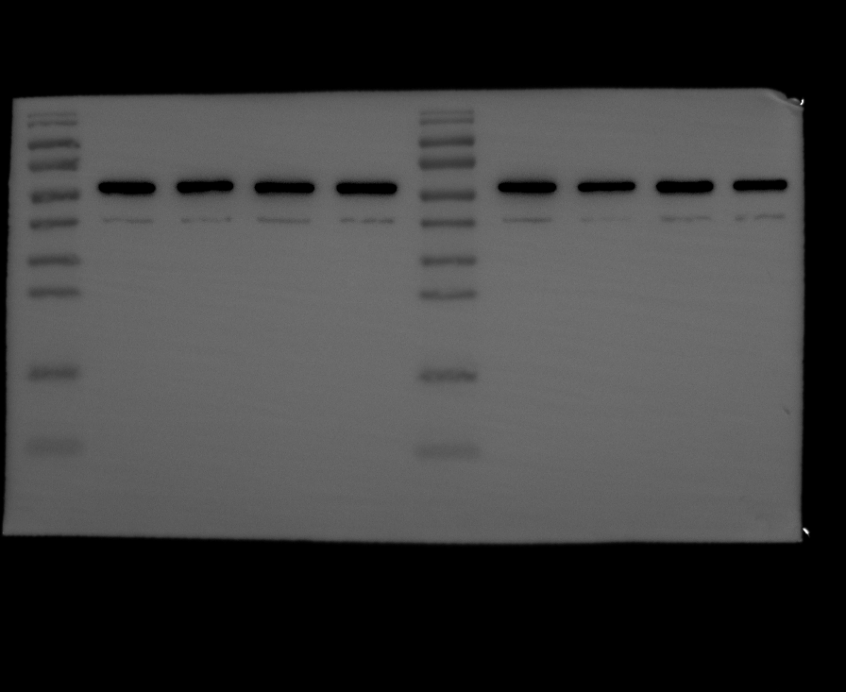


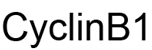


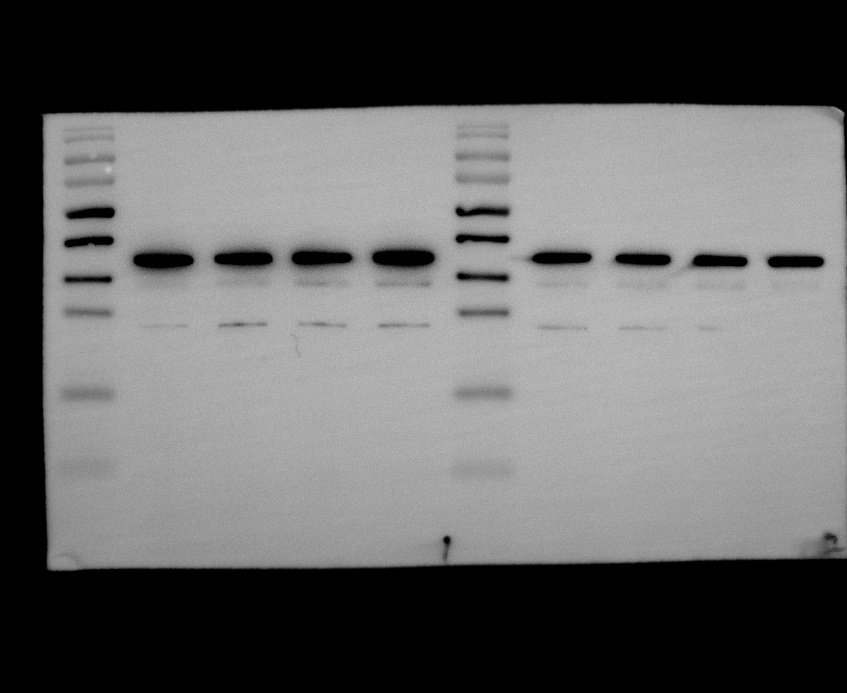

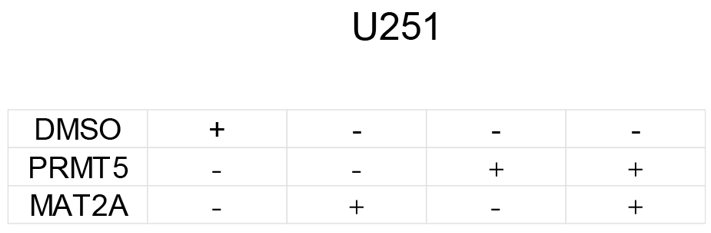

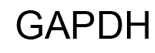


**Figure4:**


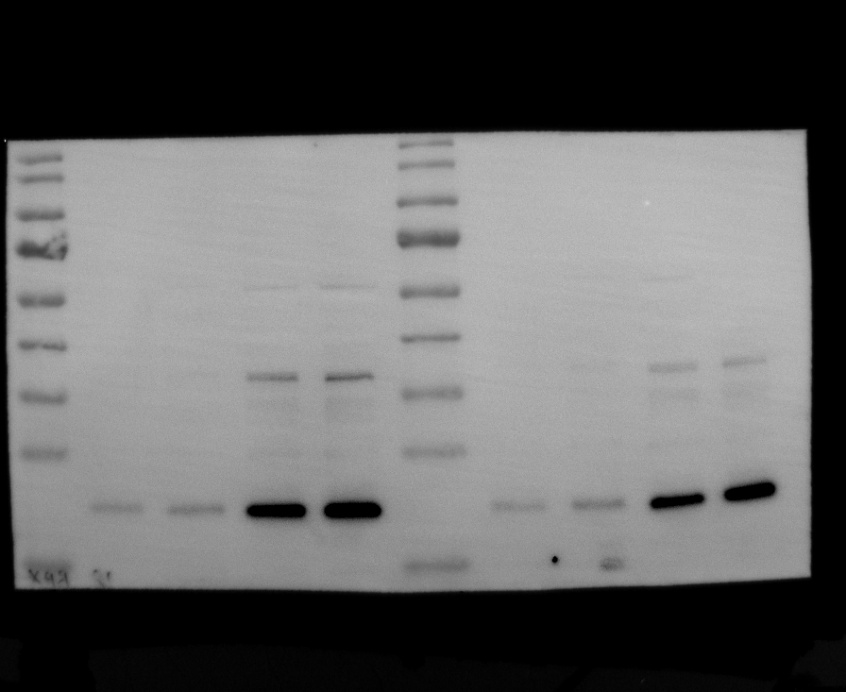

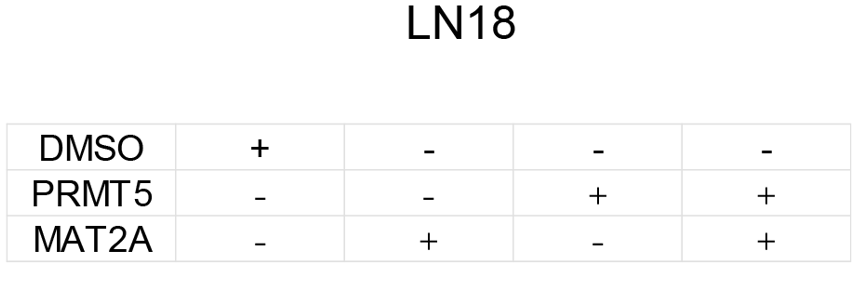


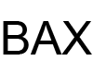


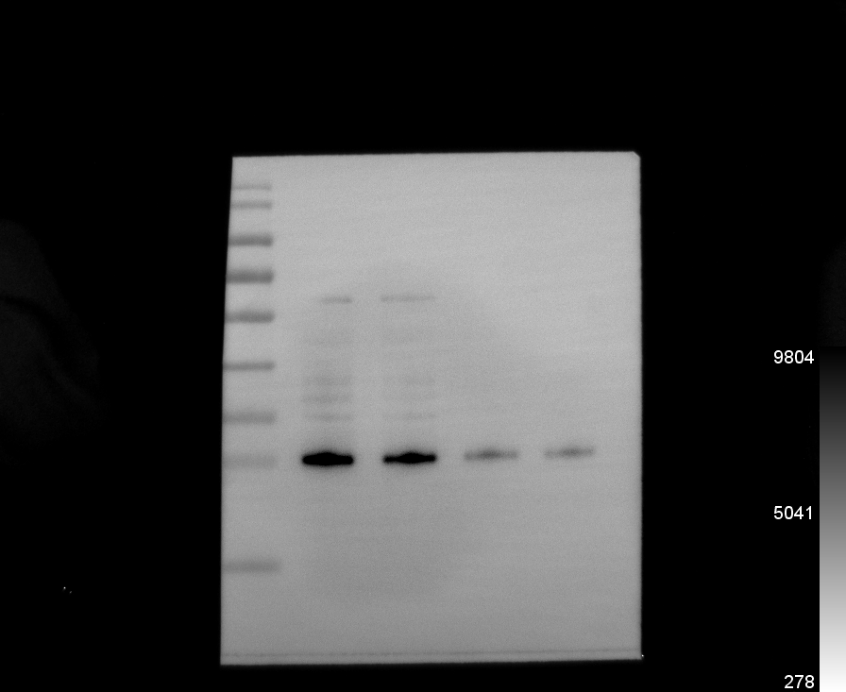

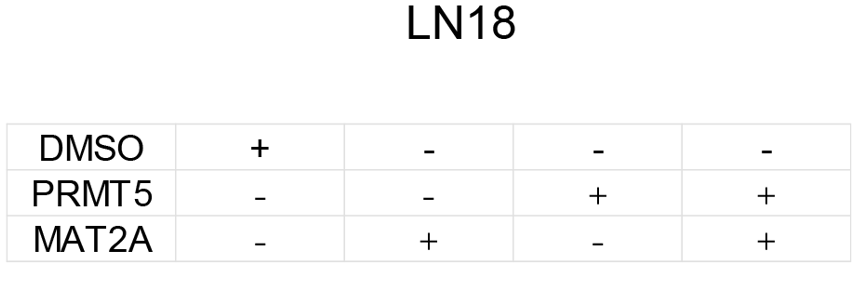


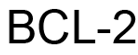


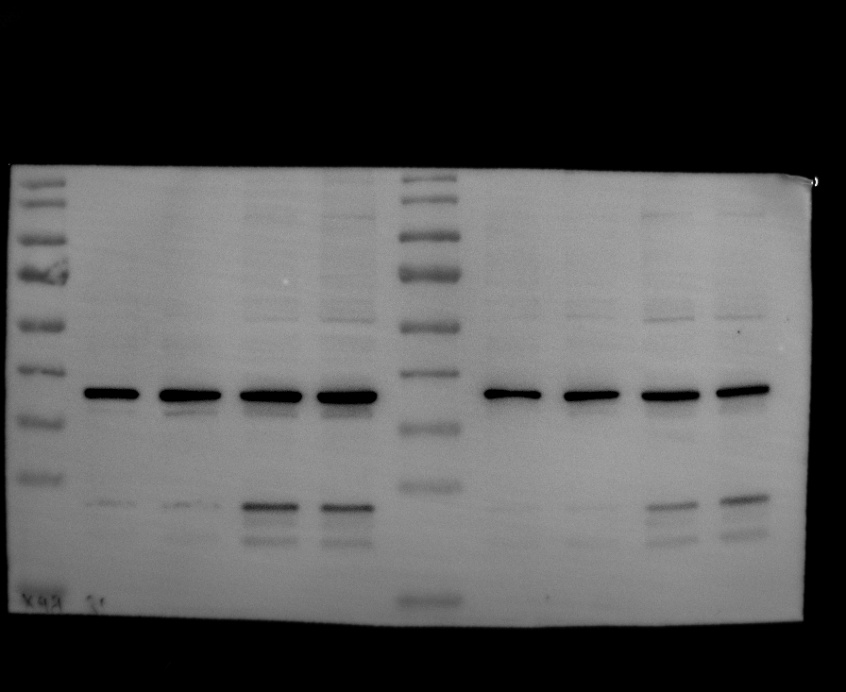

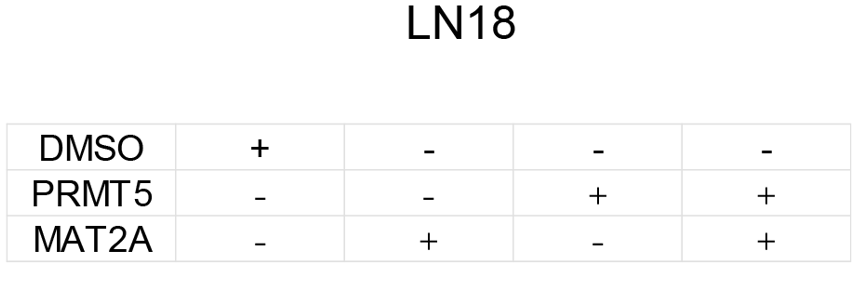

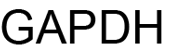


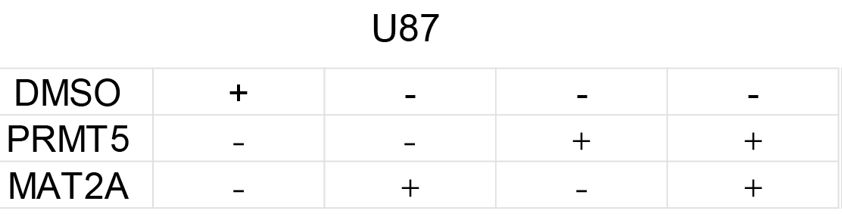


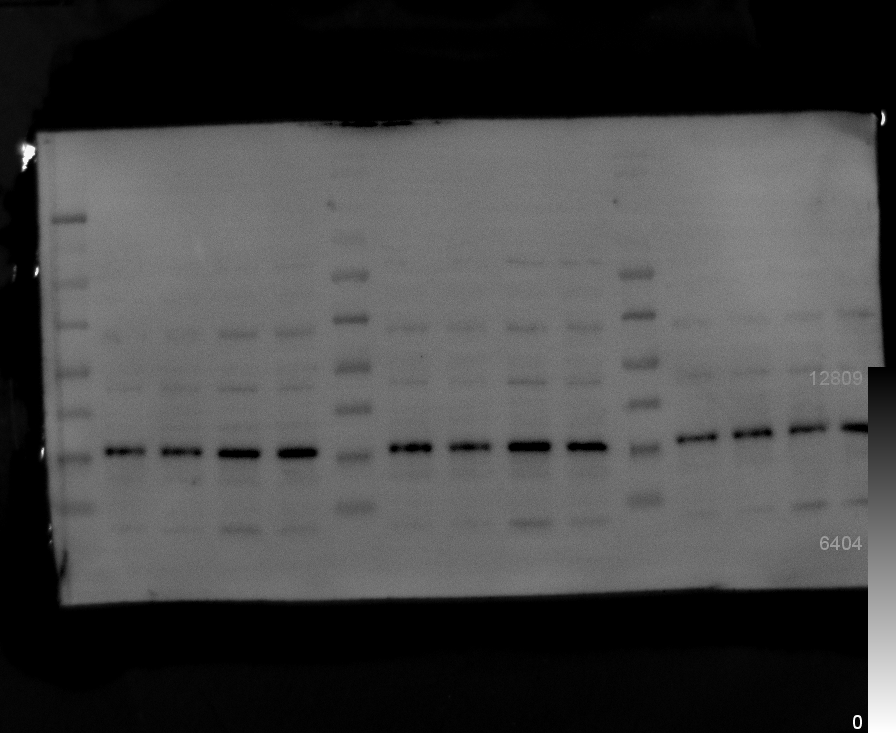


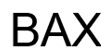


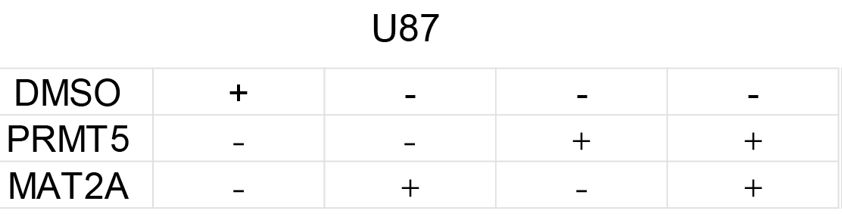


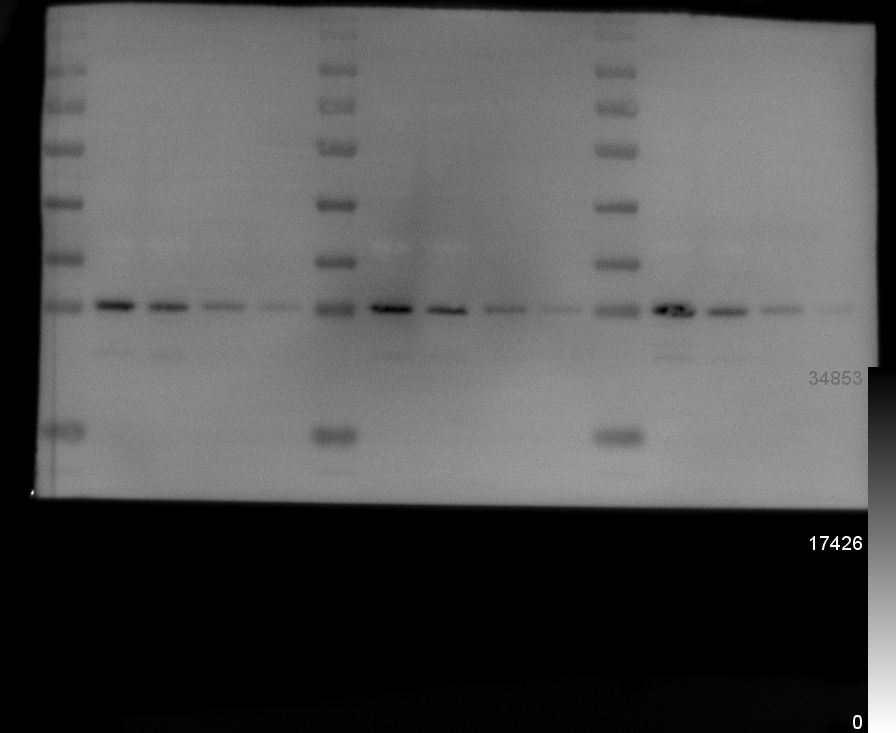


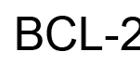


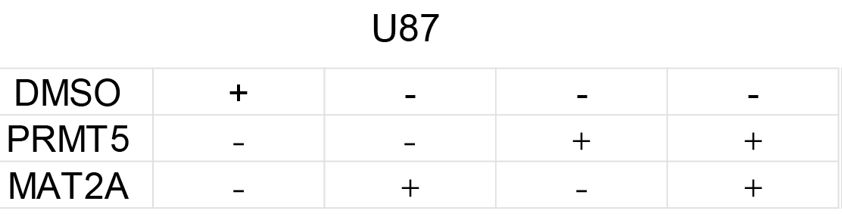


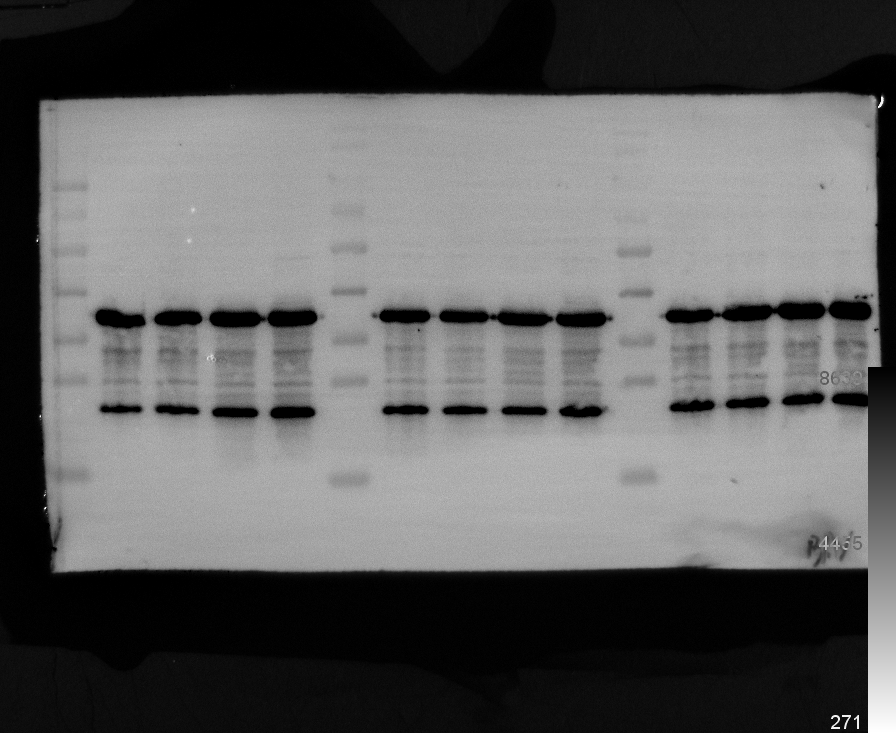


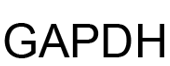


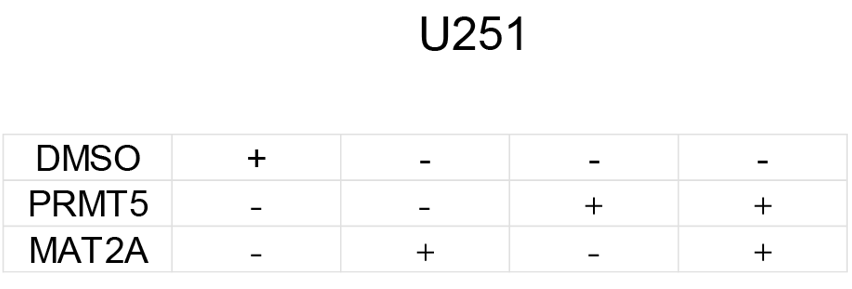


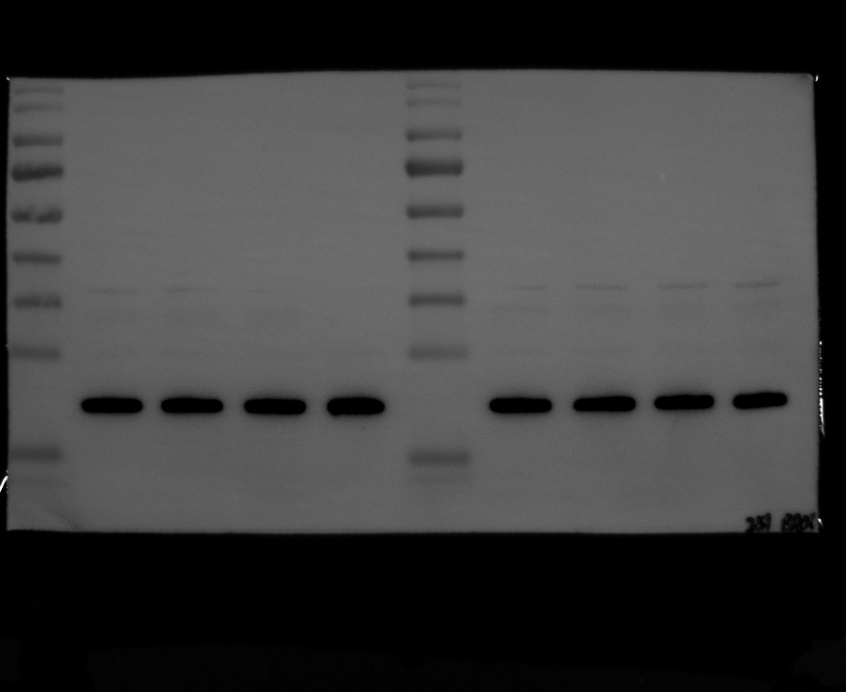


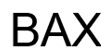


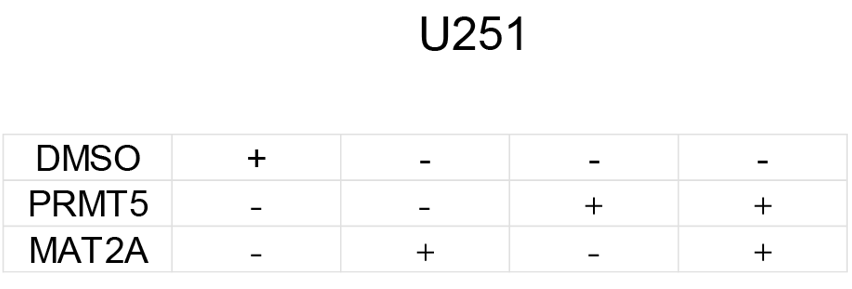


**Supplementary Figure 1**

Supplement: Supplementary file 5 — Original Data [file 41420_2025_2545_MOESM5_ESM.doc]
